# Vaccines combining slow release and follicle targeting of antigens increase germinal center B cell diversity and clonal expansion

**DOI:** 10.1126/scitranslmed.adw7499

**Published:** 2025-06-18

**Authors:** Kristen A. Rodrigues, Yiming J. Zhang, Jonathan Lam, Aereas Aung, Duncan M. Morgan, Anna Romanov, Laura Maiorino, Parisa Yousefpour, Grace Gibson, Gabriel Ozorowski, Justin R. Gregory, Parastoo Amlashi, Richard Van, Maureen Buckley, Andrew B. Ward, William R. Schief, J. Christopher Love, Darrell J. Irvine

**Affiliations:** 1 Koch Institute for Integrative Cancer Research, Massachusetts Institute of Technology; Cambridge, MA 02139 USA.; 2 Harvard-MIT Health Sciences and Technology Program, Institute for Medical Engineering and Science; Massachusetts Institute of Technology, Cambridge, MA 02139 USA.; 3 Ragon Institute of Massachusetts General Hospital, Massachusetts Institute of Technology and Harvard University; Cambridge, MA 02139 USA.; 4 Consortium for HIV/AIDS Vaccine Development, The Scripps Research Institute; La Jolla, CA 92037 USA.; 5 Department of Biological Engineering, Massachusetts Institute of Technology; Cambridge, MA 02139 USA.; 6 Department of Chemical Engineering, Massachusetts Institute of Technology; Cambridge, MA 02139 USA.; 7 Department of Integrative, Structural and Computational Biology, The Scripps Research Institute; La Jolla, CA 92037 USA; 8 Department of Immunology and Microbiology, The Scripps Research Institute, La Jolla, CA, 92037, USA; 9 IAVI Neutralizing Antibody Center, The Scripps Research Institute, La Jolla, CA, 92037, USA; 10 Department of Materials Science and Engineering, Massachusetts Institute of Technology; Cambridge, MA 02139 USA.; 11 Howard Hughes Medical Institute; Chevy Chase, MD 20815 USA.; 12 Current address: Department of Immunology & Microbiology, The Scripps Research Institute, La Jolla, CA 92037, USA

## Abstract

Vaccine adjuvants play important roles in shaping the humoral response to immunization. Here, we analyzed mechanisms of action of a clinically relevant combination adjuvant strategy, where phosphoserine (pSer)-tagged immunogens bound to aluminum hydroxide (alum) adjuvant, promoting prolonged antigen release to draining lymph nodes, are combined with a saponin nanoparticle adjuvant termed SMNP, which alters lymph flow and antigen entry into lymph nodes. When employed with a stabilized human immunodeficiency virus (HIV) envelope (Env) trimer antigen in mice, this combined adjuvant approach promoted substantial enhancements in germinal center and antibody responses relative to either adjuvant alone. Using single cell RNA and B cell receptor (BCR) sequencing, we found that the alum-pSer/SMNP combination augmented the clonal expansion and diversity of the germinal center B cell repertoire, coincident with an increased proportion of S-phase germinal center B cells and expression of positive selection markers. Moreover, we found that the combination adjuvant approach, but not alum-pSer delivery or SMNP alone, promoted accumulation of intact antigen on follicular dendritic cells, reflecting integrated effects of slow antigen delivery and altered lymph node uptake. Genetic ablation of *Cr1/2* expression by follicular dendritic cells eliminated antigen accumulation and hampered the antigen-specific germinal center response, supporting antigen delivery to these cells as a key mechanism of the improved response elicited by this combination adjuvant. These results demonstrate how adjuvants with complementary mechanisms of action impacting vaccine biodistribution and kinetics can enhance humoral immunity.

## One Sentence Summary

A vaccine combining alum-anchored antigen and saponin adjuvant increases clonal expansion and clonal diversity of germinal center responses in mice.

## INTRODUCTION

Vaccination elicits robust pathogen-specific protection against infection by prompting the immune system to recognize potential threats (1). Most licensed vaccines are thought to protect through antibody responses (1, 2), whereby antigen-specific helper T cells and B cells are activated and work together in germinal centers (GCs) to generate high-affinity antibody-secreting cells (ASCs) and memory B cells (MBCs) (3). Despite the success of vaccination-induced immunity against many pathogens, a number of major challenges remain, such as the development of effective vaccines against human immunodeficiency virus (HIV) and tuberculosis, “universal” vaccines for influenza that could provide cross-seasonal protection, or pan-coronavirus vaccines (4–7).

HIV is a useful exemplar of challenges common to these “difficult” vaccine cases. A protective vaccine will likely need to elicit several classes of broadly neutralizing antibodies (bnAbs), which recognize conserved sites on the viral envelope (Env) across the diversity of circulating viral strains. HIV-infected humans can generate bnAbs, and many classes of bnAbs have been isolated from these individuals (8, 9). However, HIV bnAbs have uncommon features such as extensive somatic hypermutation (SHM), improbable mutations, and very long complementarity-determining region 3 (CDR3) junction lengths (10, 11). Consequently, bnAb-precursor B cells are typically rare and present at very low frequencies in the human B cell repertoire (12–14).

To overcome these challenges, vaccine regimens capable of recruiting rare B cell clones into the GC reaction and promoting their expansion and affinity maturation may be required. One strategy to modulate the GC response is by manipulating the timing of inflammatory cue or antigen delivery to draining lymph nodes (dLNs). For example, sustained vaccine delivery over a few weeks using repeated injections or implantable osmotic pumps has been shown to increase the number of unique clones recruited to GCs and greatly increase the size of the GC response compared with traditional bolus vaccine administration (15–17). To make this approach more clinically translatable, we converted the most common clinical adjuvant, aluminum hydroxide (alum), into a slow-release vehicle by modifying immunogens with short phosphoserine (pSer) peptide tags (18–20). Through a ligand exchange reaction between phosphate and hydroxyls, these pSer tags anchor antigens to the surface of alum particles. This approach, which we refer to hereafter as “alum-pSer,” multimerizes the antigen on alum particles, provides greatly increased retention of the antigen bound to alum, and leads to prolonged antigen drainage from the injection site following bolus injection. These properties of alum-pSer translated into improved GC B cell and serum IgG antibody responses and the development of long-lived bone marrow plasma cells in mice for HIV and SARS-CoV-2 antigens (18–20).

A second approach to tune GC responses is through the selection of appropriate adjuvants, which can impact many aspects of the immune response including antigen presentation, immune cell recruitment and retention in dLNs, and inflammatory cytokine production that direct the adaptive immune response (21, 22). Saponins are potent adjuvants for promoting humoral responses and are used in the licensed Shingrix and Mosquirix vaccines from Glaxo-Smith Kline as well as the Novavax SARS-CoV-2 vaccine (23, 24). We recently developed a saponin-based adjuvant called SMNP, an approximately 40 nm diameter nanoparticle formed by the self-assembly of phospholipids, cholesterol, saponin, and the toll-like receptor (TLR)-4 agonist monophosphoryl lipid A (MPLA) (25). SMNP co-administration has multiple effects on the immune response in both mice and non-human primates, including enhanced lymph trafficking of antigen, increased antigen entry into dLNs, and induction of a cascade of inflammatory cytokines and chemokines in dLNs (17, 25–27). Based on these promising findings, SMNP has entered first-in-human testing through the HIV Vaccine Trials Network (HVTN144).

Inspired by their complementary mechanisms of action, we previously tested the impact of combining pSer-tagging of antigens for alum anchoring and sustained antigen release with co-administration of SMNP. We discovered that this combination adjuvant approach promoted striking amplification of humoral responses to both HIV Env and SARS-CoV-2 antigens (19, 20). Alum-pSer/SMNP immunization led to enhancements in GC B cell and T follicular helper cell (Tfh) responses and also increased serum IgG and neutralizing antibody responses. Here, we sought to investigate the immunological basis of these improved humoral immune responses and identify underlying mechanisms that might explain the potency of the alum-pSer/SMNP combination.

## RESULTS

### The combination of alum-pSer and SMNP adjuvants improves humoral immune response

**Fig. 1.**
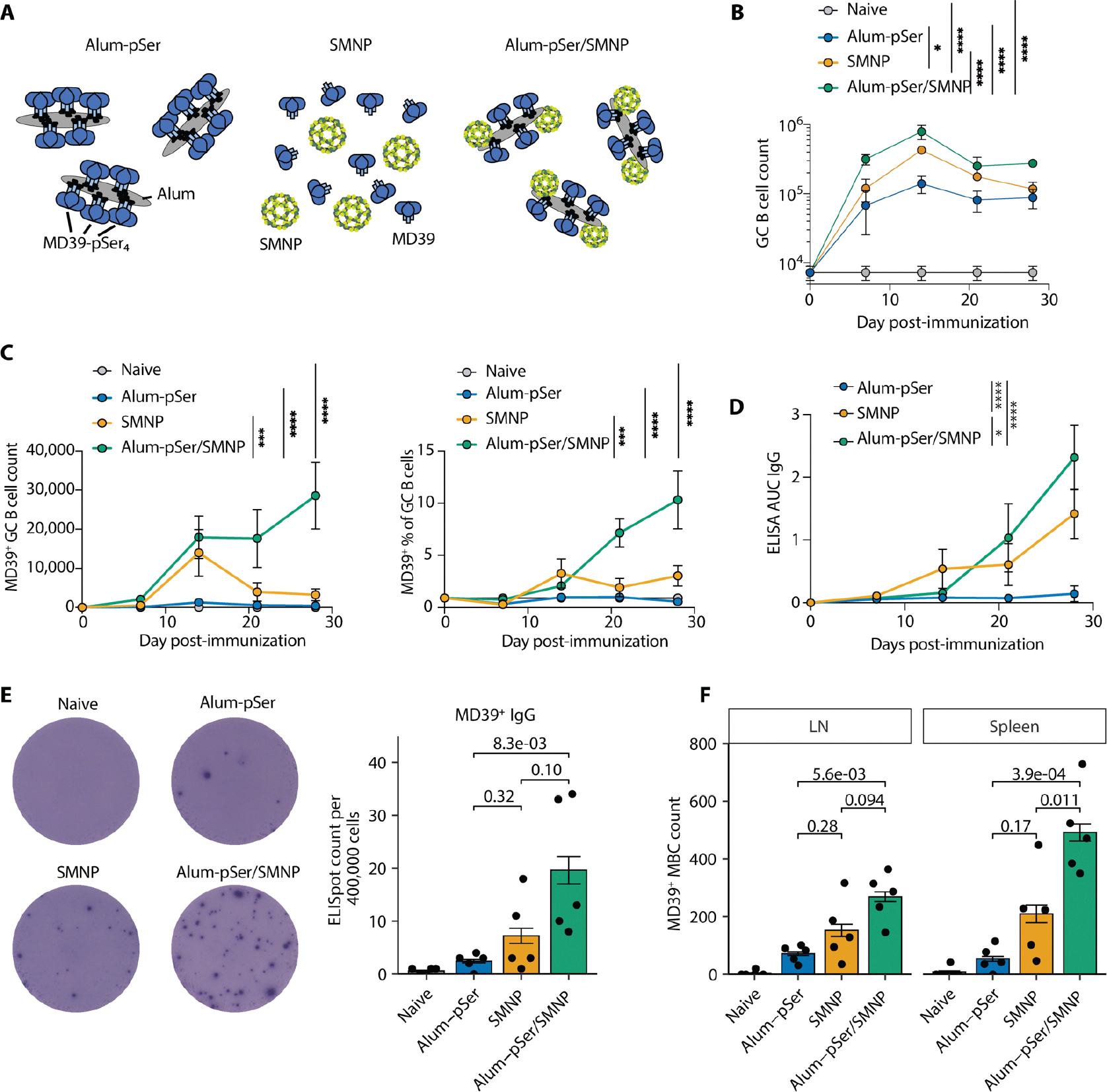
pSer-modified Env trimer anchored on alum combined with SMNP adjuvant amplifies humoral immune responses. (**A**) Schematic of immunization groups. For (B to D), BALB/c mice (*n*=5 per group for flow cytometry analysis) were immunized with 5 μg MD39 Env trimer ± 50 μg alum ± 5 μg SMNP. (**B**) Total inguinal dLN GC B cell counts over time. (**C**) MD39^+^ antigen-specific GC B cell counts and percentage among GC B cells over time. (**D**) Serum IgG antibody responses were assessed longitudinally by ELISA using MD39 captured by lectin. Values plotted are ELISA area under the curve (AUC) mean values ± s.d. Statistical significance for (B to D) was determined by two-way ANOVA followed by Tukey’s multiple comparisons test. ns p>0.05, * p<0.05, ** p<0.01, *** p<0.001, **** p<0.0001. **(E)** MD39-specific ASCs from bone marrow were assayed by ELISpot 5 weeks post-immunization. Representative wells are shown on the left, with quantification on the right. Values are mean ± s.e.m. **(F)** Bar plots of splenic and inguinal dLN MD39-specific MBC (CD73^+^PD-L2^+^) counts 5 weeks post-immunization. For (E) and (F), p values were computed by Kruskal-Wallis analysis of variance followed by Dunn’s post hoc test. For (B, C, E, and F), values plotted are mean values ± s.e.m.

To gain insights into how alum-pSer and SMNP impact vaccination, we first carried out studies evaluating how these adjuvants alone or in combination impacted humoral responses to an HIV Env stabilized SOSIP trimer immunogen termed MD39 (28). We compared three formulations (Fig. 1A). For the first vaccine formulation, a peptide tag containing four pSer residues was conjugated to the C-terminus of each MD39 gp140 protomer, leading to three pSer_4_ tags placed at the base of each trimer. When mixed with alum adjuvant, the phosphoserines of these tagged trimers undergo a ligand exchange reaction with hydroxyl groups on the surface of alum, anchoring the immunogen in an oriented fashion to the alum particles (18–20) (alum-pSer, Fig. 1A). The second vaccine formulation was comprised of MD39 trimer mixed with SMNP adjuvant (SMNP, Fig. 1A). SMNP is composed of approximately 40 nm particles of saponin, MPLA, lipids, and cholesterol, which self-assemble to form a cage-like structure; SMNP does not interact with the MD39 trimer in solution (fig. S1). The third formulation combined pSer-tagged MD39 bound to alum and SMNP particles (alum-pSer/SMNP, Fig. 1A).

Examining a single time point (day 14), we previously found that MD39 + alum-pSer/SMNP elicited antibody and GC B cell responses substantially superior to either alum-pSer or SMNP alone (18, 20). To more thoroughly assess how these adjuvants affect the GC response, we first immunized BALB/c mice with each formulation and analyzed humoral responses over time (Fig. 1B to D). We found that GC responses in all three groups steadily expanded for two weeks post-immunization, peaking at day 14, and then began contracting (Fig 1B). Consistent with our prior findings, there was a clear hierarchy in size of the GC responses, with the combination alum-pSer/SMNP immunization eliciting 1.9-fold and 5.6-fold more GC B cells than SMNP or alum-pSer alone, respectively, at the peak of the response. Further, whereas the MD39 trimer-specific GC B cell response peaked at day 14 for the alum-pSer and SMNP only groups (both in terms of absolute counts of MD39 trimer-specific GC B cells and frequencies of trimer-specific cells among GC B cells), alum-pSer/SMNP led to trimer-specific GC responses that continued expanding through 28 days (Fig. 1C). Serum antibody responses were also the strongest in the combination adjuvant group over time (Fig. 1D). Additionally, we evaluated vaccine responses beyond the GC by characterizing the formation of long-lived bone marrow ASCs and MBCs 5 weeks post-immunization finding that animals immunized with the alum-pSer/SMNP combination exhibited significantly higher counts of bone marrow ASCs than alum-pSer (*P* = 0.008; Fig. 1E). Flow cytometric analysis also revealed that mice immunized with alum-pSer/SMNP also developed a greater quantity of MD39-specific MBCs in the spleen than the individual adjuvants alone (Fig. 1F, fig. S2A). Thus, combining the alum-pSer slow-release approach with SMNP enhanced multiple facets of the humoral response to this stabilized Env trimer immunogen.

### Transcriptomic profiling of antigen-specific GC B cells primed by alum-pSer and SMNP immunizations

**Fig. 2.**
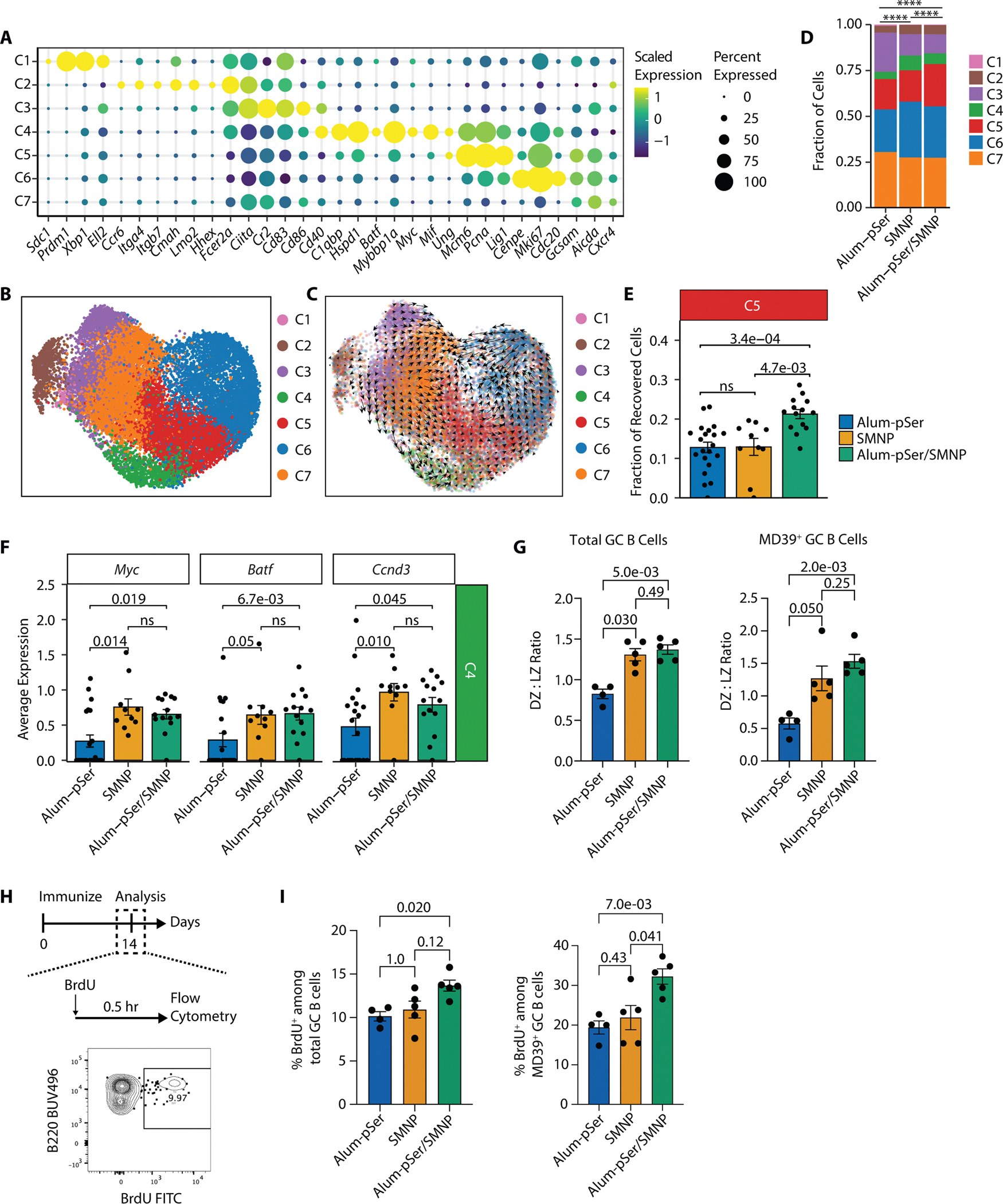
Combining Alum/pSer delivery with SMNP adjuvant promotes more GC B cells to undergo cell division. BALB/c mice (*n*=29, 14, 14 for alum-pSer, SMNP, and alum-pSer/SMNP, respectively) were immunized with 5 μg MD39 Env trimer ± 50 μg alum ± 5 μg SMNP. **(A)** Differentially expressed genes associated with phenotypic clusters. The color indicates scaled expression values. The dot radius indicates the fraction of cells expressing the gene. **(B)** Uniform manifold approximation and projection (UMAP) of phenotypic clusters of MD39-binding GC B cells. **(C)** UMAP projection of RNA velocity vector fields. The length of the arrows indicates the speed of differentiation. **(D)** Cluster distribution of recovered cells. The p values were computed by Chi-squared tests followed by Bonferroni correction. **** p<0.0001. **(E)** The fraction of C5 cells per mouse for each vaccine group. **(F)** The mouse-level average expression of *Myc*, *Batf*, and *Ccnd3* among C4 cells. For (G to I), BALB/c mice (*n*=5 per group for flow cytometry analysis) were immunized with 5 μg MD39 Env trimer ± 50 μg alum ± 5 μg SMNP. **(G)** Flow cytometry analysis of DZ:LZ ratio among total GC B cells and MD39-binding GC B cells on day 14 post-immunization. **(H and I)** S-phase cells undergoing DNA replication were labeled with BrdU by i.p. injection 30 min before euthanizing the animals and analysis of dLNs by flow cytometry. **(H)** Experimental timeline and representative flow cytometry plot. **(I)** Mean percentages of BrdU^+^ cells among total GC B cells and among MD39-binding GC B cells. For (E, F, G, and I), statistical significance was determined by Kruskal-Wallis analysis of variance followed by Dunn’s post hoc test. ns, not significant, and values plotted are mean values ± s.e.m.

We next sought to assess how this combination adjuvant affects GC responses in detail by single-cell RNA sequencing (scRNA-seq) and single-cell B cell receptor (BCR) sequencing (scBCR-seq) analyses at the peak of the total GC response for each group, day 14 post-immunization. Groups of mice were immunized with each of the three vaccine formulations, and the antigen-binding GC B cells were flow-sorted for combined scRNA-seq and scBCR-seq using SeqWell and B3E-seq protocols (29–31) (fig. S2B). The number of recovered cells per animal in each immunization condition reflected the magnitude of the GC response detected by flow cytometry (fig. S3A), but we combined cells collected from multiple animals to avoid undersampling (especially for the weaker alum-pSer alone adjuvant condition). After quality control, we recovered the transcriptomes of 19,537 MD39-binding GC B cells, including 6,480 from alum-pSer, 3,042 from SMNP, and 10,015 from alum-pSer/SMNP immunized mice. Leveraging unsupervised clustering and differential gene expression analysis, we identified seven phenotypic clusters (Fig. 2A and B). Among them, cluster 1 (C1) showed plasmablast gene signatures such as Cd138 *(Sdc1),* Blimp-1 *(Prdm1), Xbp1, and Ell2* (32). C2 upregulated *Ccr6*, *Hhex*, *Fcer2a*, and GC egressing markers *Itga4*, *Itgb7*, *Lmo2*, and *Cmah*, suggesting that C2 cells are likely GC-derived pre-MBCs (33–35). C3 cells expressed genes involved in antigen capture and presentation (*Ciita, Cr2*) and signaling with T cells (*Cd83, Cd86, Cd40*) (36, 37), implicating a light zone (LZ) B cell phenotype. Cell division is the hallmark of dark zone (DZ) B cells (3, 36). We demarcated DZ cells into three sub-clusters, where C5 was characterized by S phase genes (*Mcm6, Pcna, Lig1, Ung*), C6 by active cycling genes (*Cenpe, Mki67, Cdc20*), and C7 by canonical DZ markers (*Gcsam, Aicda, Cxcr4*). Lastly, C4 cells showed an intermediate phenotype between LZ and DZ based on their expression of positive-selection and early proliferation markers *Cd40, C1qbp, Hspd1, Mybbp1a, Myc, Batf, Mif*, and SHM and CSR marker *Ung* (36, 38–42), which is also a downstream target of Myc (43). Additionally, based on the high expression of Myc- and mTORC1-targeted genes (fig. S3B), the C4 cells are likely positively selected B cells. These clusters are consistent with phenotypes observed in prior studies of mouse and human GC B cells (37, 44–46) (fig. S3C to E).

To further validate our clustering, we performed RNA velocity analysis. The velocity vector fields showed a bifurcation among LZ cells (C3) towards pre-MBCs and plasmablasts (C1, C2) or transitioning back to the DZ (C4, C5, Fig. 2C). Cyclical cell division in the DZ was well-reflected by vector fields moving from C5 to C6 to C7. The pseudotemporal ordering of recovered B cells based on the latent time calculated from RNA velocity revealed a continuum of differentiation trajectories from the LZ (C3) or G1 DZ (C7) to cell division in the DZ (C5, C6) and eventual exit as plasmablasts (C1) or pre-MBCs (C2) (fig. S3F). The LZ and DZ intermediate cells (C4) spanned a wide range of latent time, with the majority having high latent time, implying longer residence in the GC and supporting they represent positively selected B cells. In summary, our transcriptional profiling is consistent with the current understanding of GC reactions (3, 36).

### Alum-pSer/SMNP combination adjuvant elicits an enrichment of S-phase GC B cells

Cells recovered from alum-pSer/SMNP-immunized mice showed substantially greater proportions of B cells in S phase compared with the other vaccine formulations (C5, Fig. 2D and E). Such an observation is of interest because the enrichment of S-phase B cells implies more cycles of cell division, which is an indication of having received stronger positive selection signals (47, 48). Positively selected B cells express *Myc*, *Batf*, *Ccnd3*, and mTOR complex 1 (mTORC1, consisting of *Mtor, Rptor, Akt1s1,* and *Deptor*), and the expression of these genes is proportional to selection signal strength (41, 42, 47–51). We observed higher expression of *Myc, Batf,* and *Ccnd3* in positively selected cells (C4) from alum-pSer/SMNP-immunized mice compared with alum-pSer alone (Fig. 2F); the target genes of Myc and mTORC1 (table S1, retrieved from (52, 53)) were also significantly upregulated in alum-pSer/SMNP group compared with the alum-pSer group (*P*_*Myc*_ *= 0.019*, *P*_*mTORC1*_ = 0.00071 fig. S3G). The SMNP group developed GC responses intermediate between the other two groups, having fractions of S-phase cells (C5) similar to alum-pSer and positive selection gene expression patterns more like alum-pSer/SMNP.

To validate these transcriptional observations, we characterized the proportion of DZ (CXCR4^+^) and LZ (CD86^+^) GC B cells by flow cytometry on day 14 post-immunization (fig. S4A). The DZ:LZ ratio of total GC B cells and MD39-specific GC B cells from the SMNP-adjuvanted groups were notably higher than alum-pSer alone (Fig. 2G), indicating that SMNP effectively expands the DZ compartment of GCs. To evaluate the rate of active cycling GC B cells, we injected thymidine analog BrdU 30 minutes before takedown on day 14 post-immunization (Fig. 2H). Inguinal dLNs were harvested, stained with anti-BrdU, and analyzed by flow cytometry (fig. S4B). The BrdU staining showed that alum-pSer/SMNP immunization elicited a significantly increased percentage of cycling MD39-specific GC B cells compared with either alum-pSer or SMNP alone (*P* = 0.007 and 0.041, respectively, Fig. 2I). Collectively, these results suggest that the combination adjuvant enabled MD39-binding GC B cells to undergo more cycles of cell division, likely due to intensified positive selection signaling.

### Combining alum-pSer slow release with SMNP augments clonal expansion and diversity

We next turned to paired heavy/light chain scBCR-seq to determine how alum-pSer and SMNP adjuvants impacted the repertoire of antigen-binding GC B cells. After quality control, we recovered 4,621 full-length heavy chain sequences from MD39-binding B cells, 8,670 full-length light chain sequences, and 2,950 paired BCR sequences; we recovered >1 paired BCR sequences from 19, 10, and 13 mice immunized with alum-pSer, SMNP, and alum-pSer/SMNP, respectively (fig. S5A). Recovered BCRs were evenly distributed across phenotypic clusters (fig. S5B), and B cells from expanded clones were enriched in S-phase (C5) and cycling (C6) cells as expected (fig. S5C). Greater proportions of GC B cells from SMNP and alum-pSer/SMNP immunized mice class switched to IgG isotypes (fig. S5D and E), a finding consistent with ELISA analysis of serum Ig isotypes assessed at day 28 (fig. S5F).

**Fig. 3.**
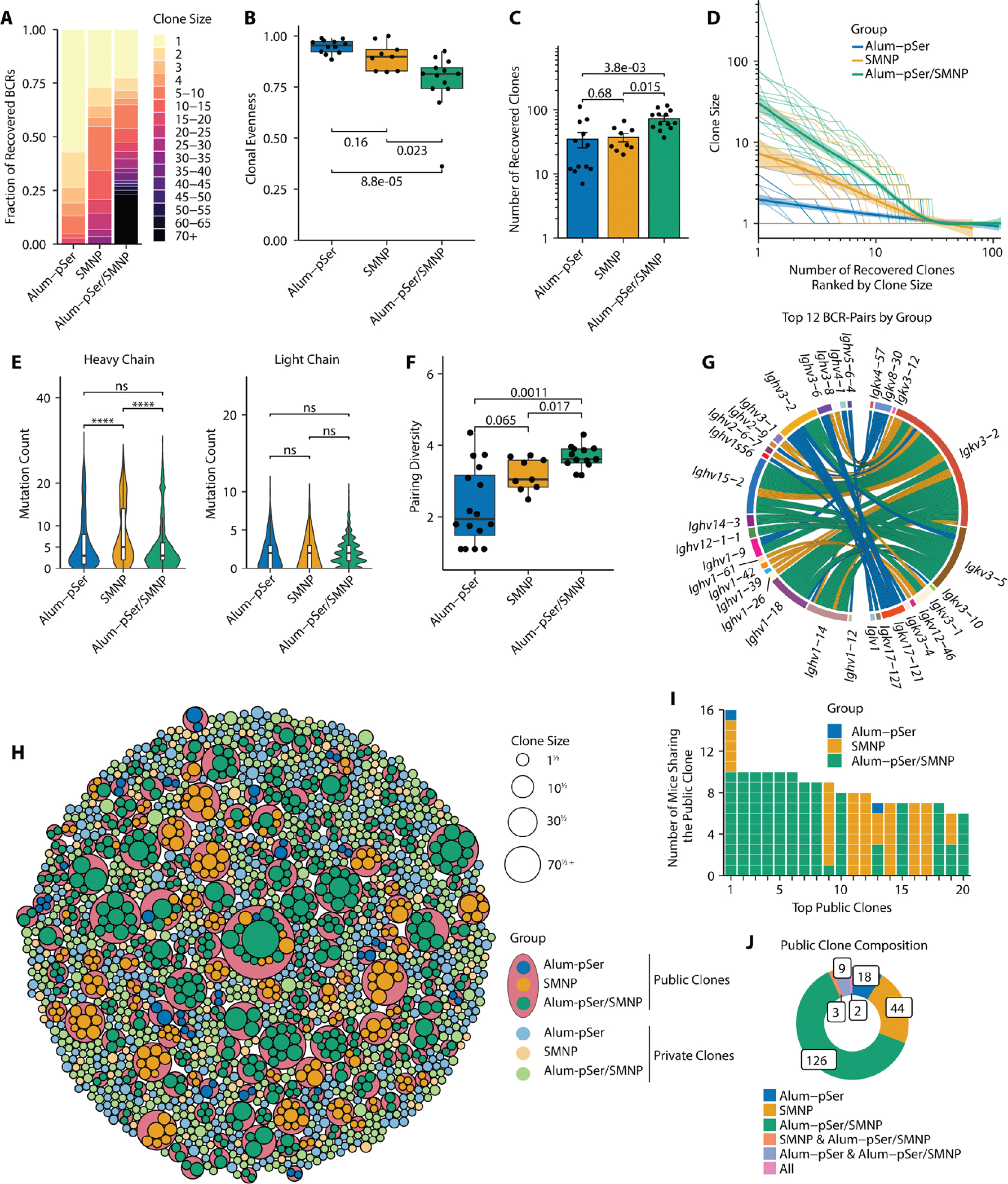
The combination of alum-pSer and SMNP adjuvants increases BCR repertoire diversity. Analysis is performed using the same dataset as Fig. 2. (**A**) Clone size distributions in each vaccine group. (**B**) The clonal evenness score computed by Pielou’s index per mouse across vaccine groups. (**C**) Number of unique clones recovered per mouse across vaccine groups. (**D**) Clone recovery curves for each mouse. Spline regression models were fit for each vaccine group. The shaded areas indicate s.e.m. (**E**) Violin plots of heavy and light chain SHM count for each recovered BCR sequence. (**F**) Clonal heavy and light chain V gene pairing diversity per mouse, calculated by the Shannon diversity index. (**G**) Top 12 common BCR pairs from each vaccine group. Each chord on the diagram represents one clone, and the colors indicate the vaccine groups. (**H**) Circle-packing diagram showing all recovered public and private clones. Private clones found in public clones (coral-colored circles) are illustrated in darker colors. Singlet private clones are illustrated as individual circles in lighter colors. The size of each circle is proportional to the square root of its clone size. (**I**) The top 20 public clones organized by the number of mice sharing that public clone. (**J**) Donut plot showing the proportion and count of different compositions of public clones. For (B, C, E, and F), p values were computed with Kruskal-Wallis analysis of variance followed by Dunn’s post hoc test. ****, p<0.0001. ns, not significant.

Clone sizes from mice immunized with the combination alum-pSer/SMNP vaccine were much larger than either individual adjuvant group, with 42 clones comprised of ten or more cells (Fig. 3A). By contrast, SMNP immunization elicited 16 clones with more than ten cells and alum-pSer elicited only 2 (Fig. 3A). As another measure of clonal expansion, we quantified clonal evenness for cells recovered from individual mice using Pielou’s evenness score (*J*) (54). This analysis revealed a significantly lower *J* score for the combination compared with SMNP (*P* = 0.023) and alum-pSer (*P* = 8.8E-05; Fig. 3B). Lower clonal evenness corroborates greater clonal expansion due to the expansion of a sub-portion of the overall clones (55). We next examined the number of unique clones in the GC and observed that the median number of primed clones per mouse was 2.2 and 3.0-fold more in alum-pSer/SMNP than SMNP and alum-pSer, respectively (Fig. 3C). Plotting recovered clonotypes from each mouse ranked by their clone sizes revealed that the combination adjuvant vaccine induced greater clonal diversity, simultaneously augmenting clonal expansion of individual clones and recruiting a greater quantity of unique clones into the GC reaction (Fig. 3D). To address sample size differences, we performed subsampling analyses by randomly sampling 600 cells from each of the formulations, calculating the mouse-level clonal evenness score and the number of recovered clones, computing the p-value by the Kruskal-Wallis Test, and repeating this process 10,000 times to generate distributions of p-values. We found that the 90% confidence intervals of p-values were (0.0040, 0.050) for clonal evenness and (0.020, 0.041) for the number of recovered clones, suggesting that the observed effect size differences were largely independent of sample size.

### Alum-pSer/SMNP vaccination enhances GC repertoire diversity

Many bnAbs for HIV have extensive SHM and require specific heavy and light chain V gene pairs (8–11, 13, 14). Therefore, vaccines that can recruit diverse B cell clones would increase the likelihood of priming rare or specific precursors capable of evolving toward broad neutralization (10, 11, 13, 16, 18). We thus sought to evaluate the SHM and the diversity of heavy/light chain V gene pairs. We first counted nucleotide mutations based on inferred germline sequences and found comparable heavy and light chain SHM for all 3 groups (Fig. 3E). This is unsurprising given prior work suggest that degrees of SHM are influenced more by elapsed time post-immunization rather than vaccine formulation (16, 17, 46). Although the SMNP group showed a statistically significant increase in heavy chain SHM counts (*P* < 0.0001), the effect size was only one nucleotide. To quantify the diversity of heavy/light chain V gene pairs, we aggregated clones by their heavy/light chain V gene usage and counted the abundance of each unique heavy/light chain V gene pair. We calculated the Shannon diversity index (*H*) for each group (56), where each unique heavy/light V gene pair represents one “species,” and the number of expanded clones using the pair represents the abundance of that species. The calculation showed increasing diversity scores in the order of alum-pSer, then SMNP, and then alum-pSer/SMNP (fig. S5G to I). The same calculation was done for each mouse, and the same hierarchy was observed; alum-pSer/SMNP showed greater BCR pairing diversity than either alum-pSer or SMNP alone (Fig. 3F). To determine whether this increase was due to the enhanced mobilization of B cells into the GC or an emergent property of the combination adjuvant, we performed subsampling analyses to compare alum-pSer/SMNP against SMNP groups by random sampling either 200 BCRs or 100 clones from each group, calculating pairing diversity scores for each mouse, performing a two-tailed Mann Whitney U test, and repeating the process 10,000 times to generate distributions of p-values (fig. S5J). Neither analysis showed statistical significance (*P* > 0.05 in both cases), suggesting that increased BCR pairing diversity in alum-pSer/SMNP was primarily correlated with overall GC size.

We observed overlaps among the most frequently used BCR pairs across the vaccine groups (e.g., *Ighv3–2—Igkv3–5, Ighv1–14—Igkv3–2, and Ighv15–2—Igkv3–2*, Fig. 3G), which motivated us to perform public clonal analyses. Public clones are similar clones of B cells found in multiple animals. The presence of public clones implies a convergent selection where BCR features are preserved across animals. We identified private clones (clones found in individual mice) and public clones (clones found in multiple mice) based on their use of (1) the same V and J genes, (2) the same HCDR3 length, and (3) a similarity threshold of their HCDR3 amino acid sequences. This analysis revealed 8.7%, 56%, and 43% clones from alum-pSer, SMNP, and alum-pSer/SMNP were public clones shared between two or more mice, respectively (Fig. 3H). Among the top 20 public clones organized by the number of mice represented, there were two public clones shared among mice from all three groups, and two more from mice receiving SMNP or alum-pSer/SMNP (Fig. 3I). 126 public clones were observed uniquely from the alum-pSer/SMNP group, 2.9 times more than SMNP-unique public clones and 7.0 times more than alum-pSer (Fig. 3J). Therefore, the combined formulation with alum-pSer/SMNP recruited more diverse antigen-specific BCRs to the GC reaction robustly across animals than the individual adjuvants alone.

To explore the conservation of our findings beyond the mouse model, we repeated our analysis on a recently published nonhuman primate (NHP) dataset, where several vaccine formulations (including alum-pSer, SMNP, and alum-pSer/SMNP) were benchmarked using the same antigen MD39 (57). To compare with the mouse model findings, we focused on analyzing samples from the earliest time point after prime immunization (week 6). Consistent with the findings in mice, NHPs immunized with alum-pSer/SMNP developed more clonally expanded GCs (fig. S6A). Following the same public clone analysis, we found that 1.8%, 20%, and 15% clones from alum-pSer, SMNP, and alum-pSer/SMNP were grouped into public clones, respectively (fig. S6B). Among the top 20 public clones, two clones were elicited by all three formulations; two included only alum-pSer/SMNP, and the rest were elicited by both SMNP and alum-pSer/SMNP (fig. S6C and D). The alum-pSer/SMNP group elicited 25 formulation-unique public clonotypes, followed by 16 SMNP-unique and 1 alum-pSer-unique public clonotypes (fig. S6D). Similar to the findings in the mouse model, this result demonstrated that many convergent BCRs were uniquely induced by the alum-pSer/SMNP adjuvant in NHPs as well. Altogether, we found that alum-pSer/SMNP formulation induced GCs with greater numbers of unique private and public clones in both mice and NHPs, suggesting that the total repertoire of GC B cells following alum-pSer/SMNP immunization is more diverse.

### Combining alum-anchored immunogens with SMNP promotes antigen trafficking to lymph node follicles

The scRNA-seq and scBCR-seq analyses showed that combining alum-pSer and SMNP adjuvants increased GC B cell clonal expansion, total number of B cell clones recruited to the GC, repertoire diversity, and increased the proportion of B cells actively undergoing cell division. We next sought to investigate mechanisms underlying these effects of alum-pSer/SMNP immunization. Heavily glycosylated antigens like HIV Env trimers, when displayed on the surface of nanoparticles, trigger complement deposition in vivo through the lectin pathway, resulting in complement-dependent trafficking of the particles to FDCs (58, 59); We hypothesized that antigen accumulation on FDCs would enable GC B cells to acquire more antigen and thus receive greater help from Tfh cells (47). The number of cell divisions in the DZ is commensurate to the strength of positive selection signals from Tfh cells (41, 42, 48, 49, 51). We hypothesized that alum particles bearing many pSer-anchored trimers per particle might trigger a similar process of antigen trafficking to FDCs, and thereby lead to expansion of proliferating GC B cells in the DZ as we observed experimentally. In parallel, SMNP triggers rapid depletion of subcapsular sinus macrophages and increases antigen accumulation in dLNs (25). These two complementary mechanisms of action may be synergistic in shepherding more MD39 antigen into the follicles and onto the FDC network. With more antigens to capture and process, GC B cells may receive stronger help from Tfh cells and undergo more cycles of cell division, which might explain the enrichment of S-phase GC B cells observed in the scRNA-seq analysis.

**Fig. 4.**
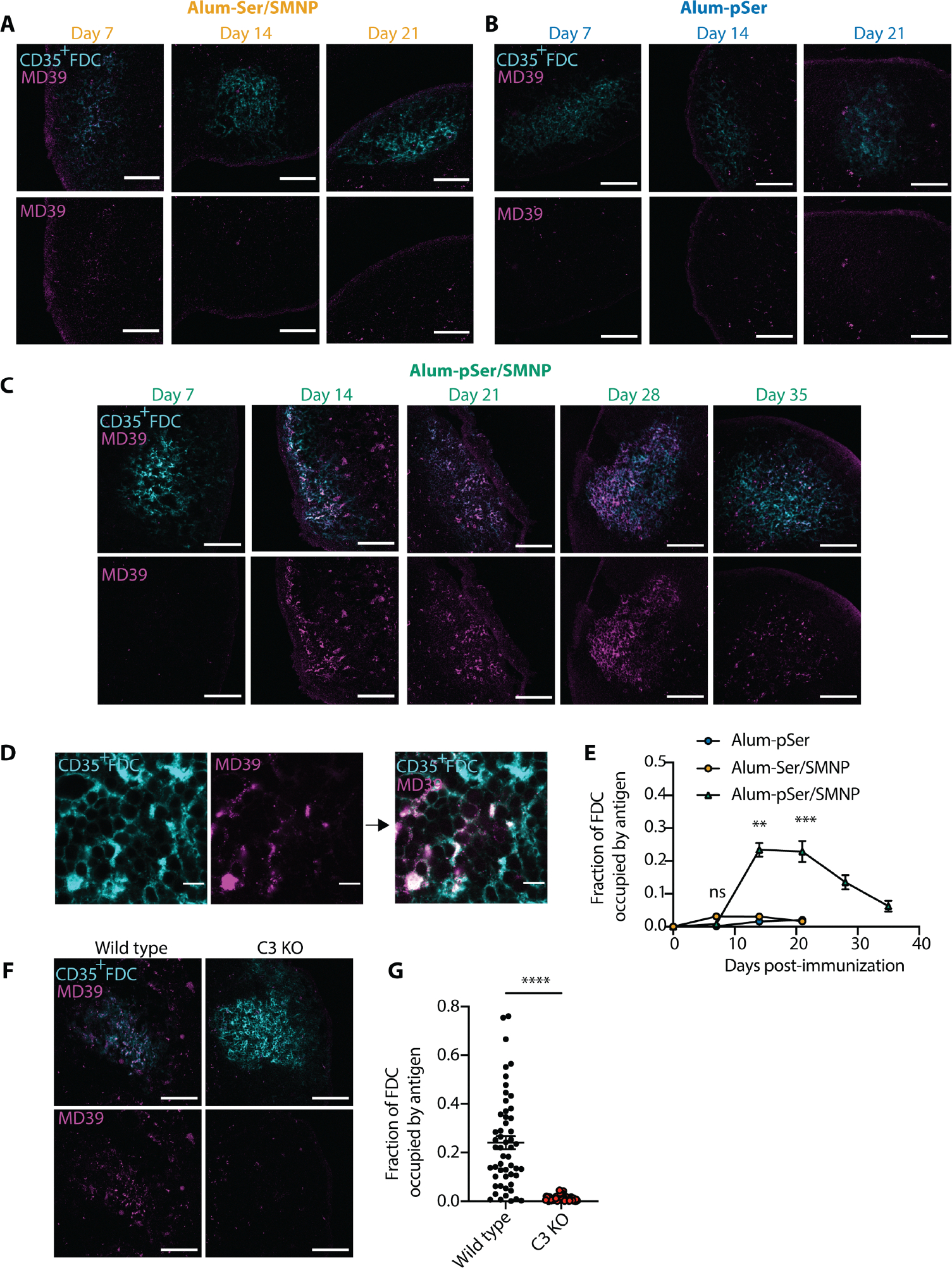
Env trimer antigen administered with alum anchoring and SMNP accumulates better on the follicular dendritic cell network over time. (**A to C**) BALB/c mice (*n*=3/group) were immunized with 10 μg fluorescently labeled MD39-Ser_4_ combined with 100 μg alum and 5 μg SMNP (**A**, alum-Ser/SMNP), MD39-pSer_4_ combined with 100 μg alum (**B**, alum-pSer), or MD39-pSer_4_ combined with 100 μg alum and 5 μg SMNP (**C**, alum-pSer/SMNP). Draining inguinal lymph nodes were isolated at the indicated time points, flash-frozen, and cryo-sectioned. Shown are representative images of antigens on the lymph node CD35^+^ FDC networks. The overlay of antigen and FDC is shown in the top row of images, with the antigen signal alone shown in the bottom row of images. Scale bars, 100 μm. (**D**) Shown are representative 100x objective lens images of CD35^+^ FDC staining and MD39 antigen, with the overlay on the right. Scale bars, 10 μm. (**E**) The fraction of FDCs occupied by antigen was calculated for each timepoint and immunization. (**F**) Wildtype C57BL/6 and C3 KO mice (*n*=3/group) were immunized with 10 μg labeled MD39-pSer_4_ combined with 100 μg alum and 5 μg SMNP. Draining inguinal lymph nodes were isolated 14 days after immunization, flash-frozen, and cryo-sectioned. Shown are representative images of antigens on the lymph node CD35^+^ FDC networks. The overlay of antigen and FDC is shown in the top row of images, with antigen signal alone shown in the bottom row of images. Scale bars, 100 μm. (**G**) Fraction of FDCs occupied by antigen for each mouse strain. Values plotted are mean ± s.e.m. Statistical significance was determined by one-way ANOVA followed by Tukey’s multiple comparisons (E) or two-tailed Mann-Whitney U test (G). ns p>0.05, ** p<0.01, *** p<0.001, **** p<0.0001.

Motivated by these ideas, we evaluated the localization of fluorescently labeled MD39 trimer in dLNs following alum-pSer and SMNP immunization. We focused on comparing antigen trafficking of alum-pSer versus alum-pSer/SMNP because we previously showed that immunization with Env trimers and SMNP alone does not lead to substantial antigen accumulation on FDCs in a primary immunization (16, 60, 61). As a control, we also assessed antigen biodistribution in dLNs for SMNP and alum mixed with MD39-Ser_4_, a trimer conjugated with a non-phosphorylated serine tag that cannot undergo ligand exchange binding to the alum particles. Mice were immunized with these three different vaccines, and inguinal dLNs were isolated at varying time points for histological imaging. Following immunization with unanchored MD39-Ser_4_ with alum and SMNP (alum-Ser/SMNP), a low amount of antigen accumulation on the FDC network was detected at day 7, but this did not persist (Fig. 4A). Alum-pSer immunization led to almost undetectable antigen accumulation on FDCs at any timepoint (Fig. 4B); both of these immunization groups showed antigen instead in the sinuses and scattered through the lymph node interior (Fig. 4A and B). By contrast, with alum-pSer/SMNP immunization, antigen accumulation was detected not only in the sinuses and dLN interior, but also strongly accumulating on dLN FDCs by day 14, where it persisted through day 35 (Fig. 4C). Higher magnification imaging showed that this antigen signal was found in B cell follicles colocalized with FDC dendrites (Fig. 4D). Quantification of the FDC-localized antigen signal from multiple follicles of multiple dLNs over time showed that following alum-pSer/SMNP immunization, antigen accumulation rose sharply between day 7 and day 14, was maintained through day 21, and then slowly decayed thereafter (Fig. 4E). This analysis revealed that the combination adjuvant vaccination elicited at least 15-fold greater antigen accumulation on FDCs compared to immunization with alum-pSer alone. To investigate the mechanistic basis of antigen accumulation on FDCs following alum-pSer/SMNP immunization, we evaluated the localization of fluorescently labeled MD39 trimer in dLNs of mice lacking the C3 component of the complement system (*C3* KO). We observed a substantial reduction in antigen colocalization with FDCs in *C3* KO mice compared to wildtype C57BL/6 mice (Fig. 4F and G), suggesting that the trafficking of antigen to the FDCs following alum-pSer/SMNP immunization is complement-dependent.

### Env trimer antigen delivered to FDCs through combined alum-pSer/SMNP vaccination is largely intact and required for optimal GC responses

Antigen localizing to the sinuses and interfollicular regions of the dLN undergoes rapid degradation over the first few days post-immunization, whereas antigens captured on FDCs can be retained in an intact state over time due to spatially compartmentalized protease activity in LNs (61). To assess the integrity of the MD39 trimer in vivo following combination alum-pSer/SMNP immunization, we applied a fluorescence resonance energy transfer (FRET)-based approach we previously developed (61). Individual MD39 antigens were labeled with approximately 6 total dyes per trimer (approximately 3 Cy3 donor and approximately 3 Cy5 acceptor dyes) for FRET imaging. When the antigen undergoes proteolytic degradation, the donor and acceptor dyes become separated, leading to reduced FRET signals proportional to the degree of degradation (fig. S7A) (61).

We first confirmed that this degree of dye labeling did not alter the alum binding behavior or antigenicity profile of the trimer (fig. S7B and C). We next tested whether the binding of dye-labeled MD39-pSer_4_ trimer to alum affected the measurement of intermolecular FRET, using an acceptor photobleaching approach to measure energy transfer. We imaged FRET dye-labeled free MD39 or alum-anchored MD39-pSer_4_ adsorbed on glass coverslips and observed an enhancement in donor emission following acceptor photobleaching indicative of FRET (fig. S7D and E). The histograms of FRET efficiencies measured pixel by pixel for free versus alum-bound trimer overlapped, indicating no impact of alum binding on the FRET signal (fig. S7F). FRET was only observed when antigens were labeled with both Cy3 and Cy5 on the same trimer, and no intermolecular FRET was observed when Cy3-labeled MD39-pSer_4_ trimers were co-loaded with Cy5-labeled MD39-pSer_4_ on alum particles, or when alum particles loaded with Cy3-labeled MD39-pSer_4_ were mixed with alum particles carrying Cy5-labeled MD39-pSer_4_ (fig. S7G). These controls indicate that FRET detected in vivo should reflect intact trimeric antigen and not intermolecular FRET between adjacent trimers loaded on alum or bound to FDCs. In addition, the FRET efficiency was not influenced by adding Cy3/Cy5-specific antibodies to the labeled trimer (fig. S7H), suggesting that potential anti-dye antibody responses, which could theoretically be elicited by dye-labeled MD39 immunization, would not alter the FRET efficiency readout.

As expected based on our previous work, a decline in FRET efficiency was observed following the incubation of FRET-labeled, alum-bound trimer with the promiscuous protease trypsin (fig. S8A and B). This correlated with a reduction in the binding of antibodies targeting the interface/fusion peptide, V3 epitopes, and CD4 binding site on the trimer as measured by ELISA (fig. S8C). Overall, these data indicated that FRET-based imaging is sensitive to detect changes in the structural integrity of the trimer.

**Fig. 5.**
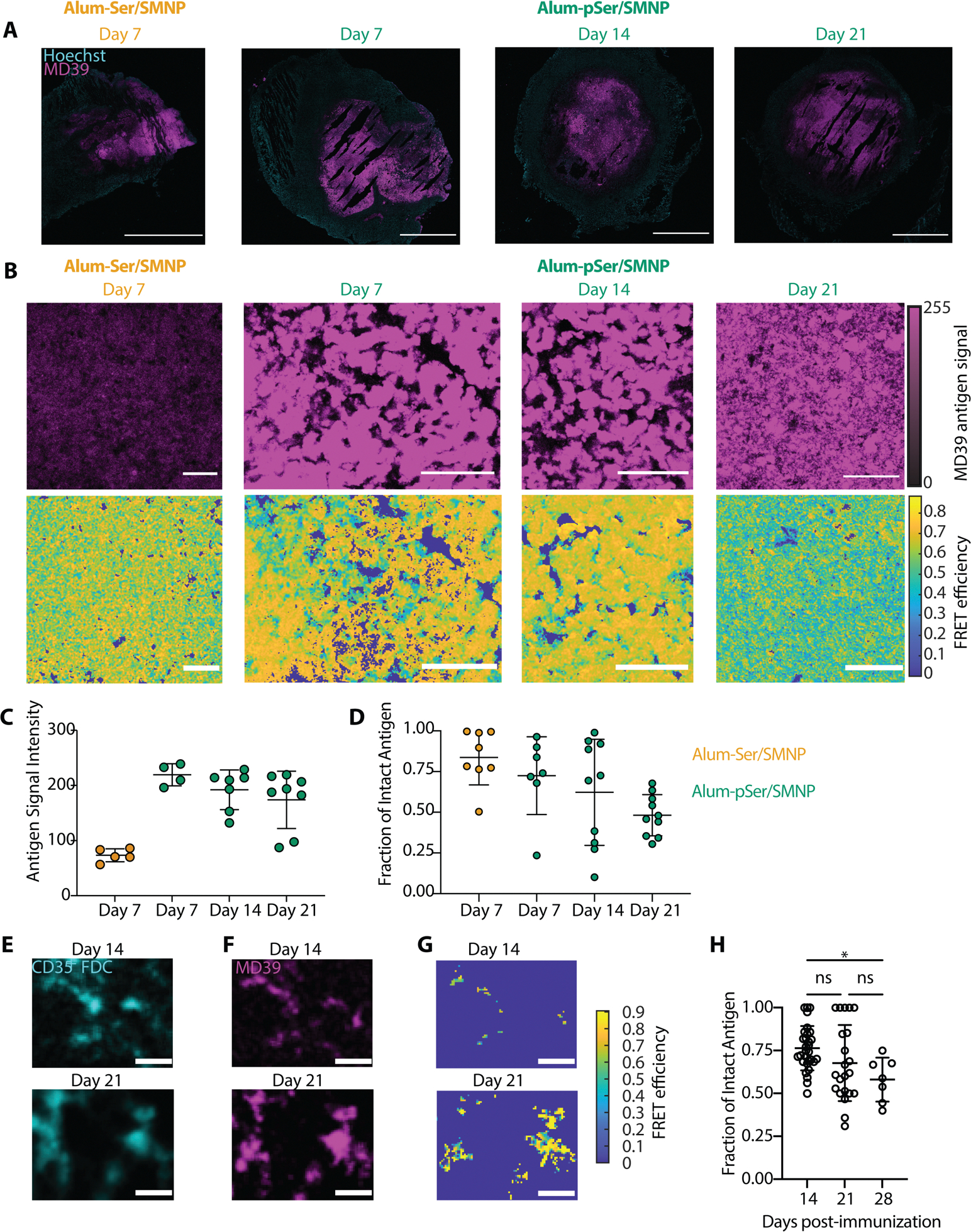
Env trimer antigen accumulated on FDCs following alum-pSer/SMNP immunization is largely intact. (**A**) BALB/c mice (*n*=3/group) were immunized with 10 μg of FRET dye-labeled MD39-Ser_4_ or MD39-pSer_4_ combined with 100 μg alum and 5 μg SMNP (alum-Ser/SMNP or alum-pSer/SMNP, respectively). Injection sites were isolated at the indicated time points, flash-frozen, and cryo-sectioned. Shown are representative images of antigen and Hoechst nuclei staining at the injection site. Scale bars represent 1000 μm. (**B**) Representative pre-bleach acceptor images (8-bit). These regions underwent acceptor photobleaching, enabling the calculation of FRET efficiencies, shown as a heatmap below. Scale bars, 50 μm. (**C**) The fluorescent antigen signal intensity of the pre-bleach acceptor images at various time points. The antigen signal intensity was calculated as the sum of the signal divided by the pixel area where the signal is nonzero. (**D**) The fraction of intact antigen at the injection site was calculated based on the antigen FRET efficiencies. (**E to G**) Representative CD35^+^ FDC (**E**) and pre-bleach acceptor images (**F**) in the draining lymph node FDC following immunization with alum-pSer/SMNP. Scale bars, 10 μm. These regions underwent acceptor photobleaching, enabling the calculation of FRET efficiencies, shown as a heatmap in (**G**). (**H**) The fraction of antigen that is intact in the follicles was calculated based on the FRET efficiencies in (G). Values in (C, D, and H) are plotted are mean ± s.d. Statistical significance in (H) was determined by one-way ANOVA followed by Tukey’s multiple comparisons test. ns p>0.05, * p<0.05.

We next evaluated trimer stability in the context of alum-pSer and SMNP immunizations in vivo. Given that alum-bound trimer is expected to slowly clear from the injection site over time, we first investigated antigen stability at the injection site. To address this, mice were immunized subcutaneously with FRET dye-labeled MD39-Ser_4_ or MD39-pSer_4_ mixed with alum and SMNP adjuvants, and injection site tissues were isolated at varying time points post-immunization. As expected, alum-anchored MD39-pSer_4_ remained detectable at the injection site through day 21, but MD39-Ser_4_ was detectable at the injection site only on day 7 and not at later time points (Fig. 5A). This drainage pattern between the two formulations is consistent with prior whole animal fluorescence imaging studies (20). Acceptor photobleaching FRET was used to quantify the total antigen present and the fraction of intact antigen at the injection site for each condition and time point (Fig. 5B). This analysis revealed much higher amounts of trimer were present at the injection site in the alum-pSer/SMNP group on day 7 compared to the alum-Ser/SMNP group employing unanchored MD39 (Fig. 5C), and, although there was a decline in antigen stability over time, approximately 50% of alum-anchored MD39 was still intact at the injection site 21 days after immunization (Fig. 5D).

In parallel, we imaged antigens accumulated on the FDC network in draining inguinal lymph nodes. The majority of the antigen that accumulated on FDCs following alum-pSer/SMNP immunization was non-degraded and remained intact through day 28 (Fig. 5E to H). Altogether, these data indicate that, unlike alum-pSer or SMNP adjuvants individually, the combination alum-pSer/SMNP immunization leads to antigen targeting to FDCs over time, and this antigen is retained in a highly intact state for at least one month post-immunization.

**Fig. 6.**
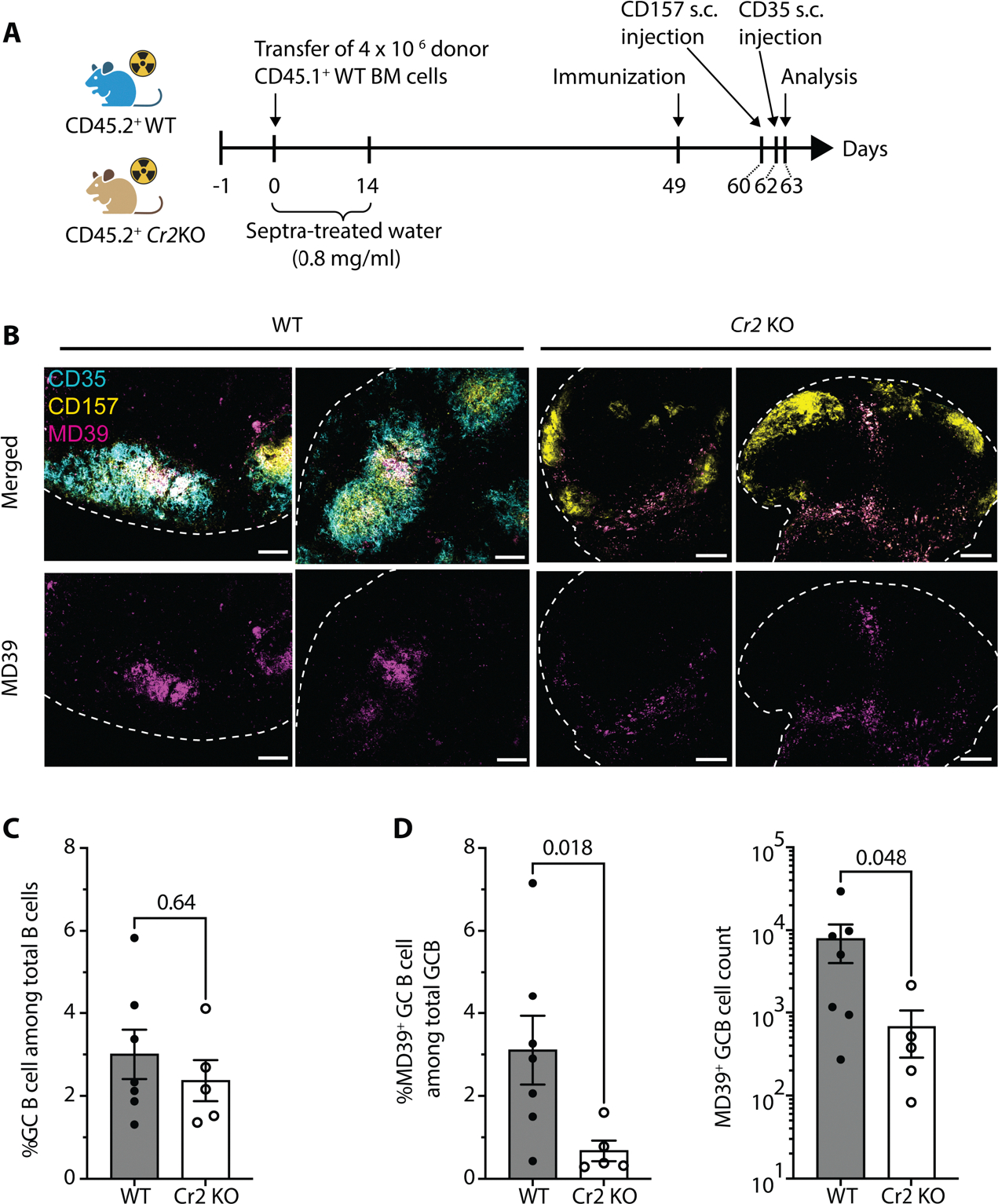
Disrupting antigen accumulation on FDCs reduces antigen-specific GC responses. **(A)** Study timeline. CD45.2^+^ WT and CD45.2^+^ *Cr2*KO mice (*n*_WT_=3 and 7, *n*_*Cr2*KO_=3 and 5 for microscopy and flow cytometry analyses, respectively) were lymphodepleted with radiation a day before reconstitution with 4×10^6^ CD45.1^+^ WT bone marrow (BW) cells. The chimera mice were housed with Septra-treated water for two weeks, immunized with MD39-pSer_4_ combined with 100 μg alum and 5 μg SMNP on week 7, and analyzed on week 9. **(B)** Fourteen days post-immunization, inguinal dLNs from BM chimeras were isolated, cleared, and imaged by confocal microscopy. Fluorescently labeled anti-CD157 and anti-CD35 antibodies were injected subcutaneously at the tail base 3 days and 1 day before the study endpoint, respectively. Shown are fluorescently labeled MD39 (magenta) and antibody staining for CD35 (cyan) and CD157 (yellow). The antigen signal alone is shown in the bottom row of images. Scale bars, 100 μm. **(C and D)** Inguinal dLNs were isolated on day 14 post-immunization, and GC responses were analyzed by flow cytometry. Shown are percentages of GC B cells out of total B cells (C) as well as percentages and absolute cell counts of MD39^+^ GC B cells (D). For (C and D), data are presented as mean ± s.e.m., and statistical significance was determined by two-tailed Mann-Whitney U tests.

To determine if this antigen capture was important for promoting the enhanced GC response achieved by alum-pSer/SMNP immunization, we evaluated the immune response elicited in animals where complement receptors (CR1/2) were deleted from FDCs. This would block antigen capture by the FDCs while maintaining CR1/2 expression on hematopoietic cells, minimally perturbing other elements of the humoral response. Bone marrow chimeras were generated by transferring wildtype (WT) bone marrow cells into irradiated wildtype C57Bl/6 or *Cr2* KO (62) recipients (Fig. 6A). The reconstitution was confirmed 4 weeks after cell transfer (fig. S9A). Seven weeks post-reconstitution, we immunized chimeras with alum-pSer/SMNP using fluorescently labeled MD39-pSer_4_ and assessed antigen localization in lymph nodes two weeks later. Prior to sacrificing the animals, fluorescent CD35 and CD157 antibodies were injected to label FDCs and ongoing GC reactions (58), respectively. Imaging of cleared inguinal dLNs revealed that WT recipients showed robust trimer accumulation on FDCs in ongoing GCs (Fig. 6B). In contrast, CD35 staining was absent from *Cr2* KO recipients, and although GCs were detected by CD157 staining, little to no antigen was detectable in these follicles. We next assessed the development of antigen-binding GC B cells. WT or *Cr2* KO chimeras were immunized with alum-pSer/SMNP, and GCs were analyzed by flow cytometry on day 14 (fig. S9B). The total size of the GC response was comparable for the two chimeras (Fig. 6C); however, antigen-specific GC B cells were reduced by approximately 5-fold by percentage and approximately 12-fold by count in *Cr2* KO chimeras (Fig. 6D). Together, these data suggest that antigen localization in follicles triggered by the slow-release/SMNP immunization is important for promoting the maturation of antigen-specific GC responses.

## DISCUSSION

Slow-release vaccine approaches promote early immune complex formation and sustained availability of antigen for B cells that together have been shown to increase the diversity of recruited clones and correlate with enhanced neutralization breadth (15–17). Meanwhile, particulate display of antigens is conducive to BCR crosslinking and B cell activation, which has been shown to facilitate the activation of B cells, restrict access to base-proximal epitopes, and cultivate the maturation of low-affinity precursors (58, 60, 63, 64). The formulation with alum-pSer enables both of these elements to be incorporated into vaccines in a simple manner. SMNP is also a potent adjuvant that has been demonstrated to enhance lymph flow, increase antigen trafficking into follicles, and triggers robust induction of inflammatory cytokines in draining lymph nodes (25, 27). These distinct (and we hypothesized, complementary) mechanisms of action underlying slow release, particulate antigen, and follicle targeting formulations inspired our initial studies examining the alum-pSer/SMNP combination. We previously analyzed immune responses to alum-pSer-delivered vaccines with or without the addition of SMNP adjuvant for relatively immunogenic antigens such as the SARS-CoV-2 receptor binding domain and an engineered germline targeting HIV gp120 immunogen eOD-GT8 (18, 19). Here we focused on analyzing the in-depth effects of this combination of adjuvants using a recently-generated stabilized native-like Env trimer MD39 that was explicitly optimized for delivery by the pSer-tagging approach (20). We found that this combination adjuvant vaccine enhanced multiple facets of the humoral immune response, including greater GC B cell, memory B cell, antibody-secreting cell, and serum IgG antibody responses. Although mice are generally unable to elicit HIV bnAb-lineage B cell responses due to their short CDR3 domains (65), we found that this combination adjuvant strategy recruited a more clonally expanded and diverse B cell response and resulted in GCs that included increased numbers of antigen-specific BCR clones elicited across animals, all features expected in humans to promote more reliable priming of rare precursor B cells that are needed for bnAb responses against HIV and other infectious diseases.

One important consideration for vaccine slow-release approaches is the possibility of antigen degradation over time following administration, either at the injection site or en route to dLNs. Such degradation could influence B cell competition and immunodominance, diverting responses toward vaccine-irrelevant breakdown product epitopes (66). In vivo, protease expression in LNs can promote rapid antigen degradation, but intriguingly, B cell follicles are a sanctuary site for the retention of intact antigens (61, 67). An important finding in this study was the discovery that alum-pSer/SMNP vaccination triggered substantial and sustained antigen accumulation on FDCs that was not observed with alum/pSer or SMNP alone. Using a FRET imaging-based approach to track antigen integrity over time, we found that a majority of alum-bound trimer delivered with SMNP remained intact for at least 28 days post-injection. We observed that the frequency of GC B cells recognizing intact trimer steadily rose over time following alum-pSer/SMNP immunization, perhaps reflecting the unique capacity of the combination adjuvant to promote intact antigen retention in follicles. Genetic deletion of *Cr1/2* from FDCs led to the loss of this antigen capture and weakened the antigen-specific GC response. This provides evidence that antigen retention by FDCs is an important contributor to the GC response elicited by alum-pSer/SMNP strategy, even when antigen is present in regions outside the follicle. Although larger GC responses were already initiated by day 7 in the alum-pSer/SMNP combination, GC B cells with sufficient affinity to be detected by antigen tetramers were only robustly expanding by day 14, and, for the combination group, they continued to expand through 28 days. This suggests that low amounts of antigen (whether localized in follicles or not) early following vaccination can initiate GC responses, but expansion of antigen-specific GC B cells requires sustained intact antigen availability in the follicles at later times.

Previous studies have demonstrated that antigen particles decorated with complement (either due to innate immune recognition or immune complex formation (68–70)), efficiently accumulate on FDCs, leading to enhanced GC and serum antibody responses (58, 60). Antigens captured by FDCs shape the B cell response, as FDCs present antigens to B cells in the follicle where activated B cells undergo proliferation and SHM to generate high-affinity antibodies (70, 71). Prior work has shown that soluble HIV trimer immunogens delivered as bolus injections predominantly localize in interfollicular regions through SIGN-R1^+^ lymph node macrophages, which capture the trimer from the afferent lymph, rather than trafficking to FDCs (58, 72). Being able to deliver antigens onto the FDC network is of interest because (i) FDCs can recycle and protect antigens captured on their dendrites (68), (ii) the follicles are sanctuary sites with low protease activity in lymph nodes where antigens are protected from proteolytic degradation (61), and (iii) we provide direct evidence in this study that expansion of “on target” GC B cells recognizing intact antigen is substantially weakened when antigen accumulation on FDCs is disrupted. It is known that protein nanoparticles or immune complexes decorated with complement are shuttled to FDCs in a complement- and complement receptor-dependent manner, mediated by noncognate B cells picking up the complement-decorated antigen and transferring it to FDCs (58, 69, 73–75). In the present case, there are at least three possible pathways for alum-pSer-trimer particles to trigger complement deposition: (i) early antibody responses elicited over the first 1 to 2 weeks could promote the formation of immune complexes as alum-pSer-trimer complexes slowly drain from the remaining injection site depot to the dLN, similar to effects observed with repeat-injection immunizations (15, 16); (ii) the alum particles may directly activate complement (76, 77) leading to C3 decoration of the alum-pSer-trimer complexes; or (iii) complement deposition could be triggered by mannose-binding lectin recognition of trimer-decorated alum particles (58). Although we show here homing of antigen to FDCs following combination adjuvant vaccination is dependent on complement, further dissecting whether one or more of these pathways governs this response remains an open question for future work. SMNP is expected to amplify this antigen delivery process by causing early depletion of subcapsular sinus macrophages that limit antigen entry into the dLN.

Our study is not without limitations. First, although our data demonstrates an important role for complement in antigen delivery to FDCs following alum-pSer/SMNP immunization, the precise mechanism of complement activation by the vaccine remains to be clarified. Second, we performed scBCR-seq at only one timepoint, the peak of GC response for all three groups. It will remain of interest for future work to examine the evolution of the BCR repertoire over time among the three formulations. Given our observation that alum-pSer/SMNP led to greater GC B cell proliferation and clonal diversity, it would be interesting to perform scBCR-seq on antigen-specific GC B cells, MBCs, and ASCs at later time points as well. Third, the mechanism behind the observation of a greater convergent BCR response following immunization with alum-pSer/SMNP compared to the individual adjuvants (Fig. 3J, fig. S6D) remains to be further studied. One hypothesis could be that alum-pSer/SMNP immunization lowers the GC selection pressure, enabling B cells with weak affinity to MD39 to develop. Therefore, B cells specific for many sub-dominant epitopes that would be eliminated early in single adjuvant groups, were preserved in the alum-pSer/SMNP group and captured by scBCR-seq on day 14. A model antigen with limited epitopes, where the epitopes’ relative immunodominance levels are known, can be employed to test this hypothesis.

In conclusion, these studies demonstrate how combining slow antigen release through immunogen anchoring on alum with a potent saponin/TLR agonist adjuvant can alter antigen biodistribution in dLNs, leading to a sustained buildup of intact antigen captured in B cell follicles. This alteration in antigen delivery correlated with substantial changes in the composition of the GC response triggered by the combination of these two adjuvants, including recruitment of a more diverse set of B cell clones to the GC that undergo greater clonal expansion compared with vaccines using either adjuvant alone. More broadly, this work reinforces the concept that strategies for vaccination designed to promote increased availability of antigen over time on FDCs can augment diverse elements of the humoral immune response.

## MATERIALS AND METHODS

### Study design

The objective of these studies was to investigate the mechanism by which the combination of alum-anchored pSer-modified HIV Env immunogens with SMNP adjuvant elicits improved humoral immune responses, as a clinically translatable approach to promote slow release of vaccine following a single injection. BALB/c mice (unless otherwise specified in the figure legend) were immunized, and subsequent humoral immune responses (germinal center responses, memory B cell, antibody-secreting cell responses, and serum antibody responses) were assessed by flow cytometry, ELISpot, ELISA, and integrated scRNA/BCR-seq. Antigen trafficking studies were completed using fluorescently labeled antigens and imaged by confocal microscopy. Antigen integrity studies were completed leveraging donor dye and acceptor dye-labeled antigens, where acceptor photobleaching was used to track antigen integrity by FRET. This acceptor photobleaching method avoids donor and acceptor crosstalk and is not influenced by dye concentrations or ratios. For sequencing studies, group sizes were selected based on pilot flow cytometry runs to determine the number of antigen-specific GC B cell counts per animal. For histology and flow cytometry studies, group sizes were selected based on power calculation using effect sizes seen in prior studies. All animals were randomly assigned to the experimental groups. The number of samples and replicates used are included in the figure legends.

### Antigen production and pSer conjugation

MD39 immunogens with or without a free C-terminal cysteine and containing a positively charged, non-polyhistidine amino acid sequence (Lys-Lys-Lys) at the C-terminus of the trimer with or without a filled glycan hole at residues N241 and N289 (20, 78, 79) were synthesized as described previously (28, 80). Briefly, genes encoding MD39 HIV Env gp140 were cloned into pHLsec by Genscript and co-transfected with human furin in a pcDNA3.1 plasmid using a 2:1 trimer:furin DNA ratio with polyethylenimine into FreeStyle 293-F cells (Thermo Fisher) and incubated for 6 days. The cultures were centrifuged and the supernatants containing MD39 were harvested and purified using a HisTrap HP column (Cytiva Life Sciences) with an AKTA FPLC system (Cytiva Life Sciences) for immunogens expressed with a polyhistidine linker and a 2G12 immunoaffinity column for MD39 immunogens without a polyhistidine linker. The immunogens were further purified by size-exclusion chromatography with an S200 Increase column (Cytiva Life Sciences) in TBS at flow rate of 0.5 mL/min. Size exclusion chromatography multi-angle light-scattering (SECMALS, DAWN HELEOS II and Optilab T-rEX Wyatt Technology) was then used to confirm the trimer molecular weights.

Immunogens expressed with a free terminal cysteine were reduced at 1 mg/mL with 10 molar equivalents of tris(2-carboxyethyl)phosphine (TCEP, Thermo Fisher) in tris-buffered saline (TBS) and incubated at 25°C for 10 minutes. TCEP was subsequently removed from the reduced protein solution using Amicon Ultra Centrifugal Filters (10 kDa MWCO, Millipore Sigma) in TBS (Sigma Aldrich), and 1 mg/mL reduced antigen was reacted with 5 molar equivalents of Ser_4_-maleimide or pSer_4_-maleimide linkers for 16 hours at 4°C in TBS (pH 7.2–7.4). Free peptide linker was subsequently removed using 10 kDa MWCO centrifugal filters in TBS, and pSer-antigen was buffer exchanged to phosphate-buffered saline.

pSer_4_-conjugated cytochrome C used for antigenicity profiling of immunogens was prepared as described (18), using cytochrome C from *Saccharomyces cerevisiae* (Sigma Aldrich). The number of pSer residues conjugated to the antigen was assessed using the Malachite Green Phosphoprotein Phosphate Estimation Assay Kit (Thermo Scientific) against a standard curve of pSer-maleimide linker. Signal from pSer-antigen was compared to the background from an unconjugated antigen control.

### Animals and immunizations

Experiments and handling of mice were conducted under federal, state, and local guidelines under an Institutional Animal Care and Use Committee (IACUC)-approved protocol. Female 6–8-week-old BALB/c, C57BL/6, *C3* KO (81), *Cr2* KO (62) mice were purchased from the Jackson Laboratory (stock no. 000651, 000664, 029661, and 008225, respectively). To generate bone marrow chimeras, *Cr2* homozygous KO and wildtype C57BL/6 littermates were irradiated twice at 475 cGy 4 hours apart and injected with 4 million donor CD45.1 WT bone marrow cells 24 hours later. Mice were housed with Septra-treated water (0.8 mg/ml) for two weeks. Reconstitution was confirmed 4 weeks post-cell transfer by flow cytometry. Immunizations were conducted 7 weeks post-cell transfer and analyzed at 14 days post-immunization. For cleared dLN imaging of bone marrow chimera mice, fluorescently labeled monoclonal antibodies against CD35 (4 μg, clone 8C12, BD Biosciences # 740029) and CD157 (20 μg, clone BP-3, BioLegend #140204) were injected s.c. at the tail base 24 and 72 hours before tissue collection, respectively, for in situ labeling of FDCs and ongoing GCs, respectively.

Immunizations for sequencing studies were prepared by mixing 5 μg of antigen with a glycan hole at residues N241 and N289 and 50 μg of alum in 100 μL sterile TBS (Sigma Aldrich) per mouse. Immunizations for histology, flow cytometry, and FRET studies were prepared by mixing 10 μg of antigen with a filled glycan hole at residues N241 and N289 and 100 μg of alum in 100 μL sterile TBS (Sigma Aldrich) per mouse. Antigen was loaded onto alum for 30 minutes on a tube rotator before immunization. For alum-pSer/SMNP combination vaccines, antigen was first loaded onto alum for 30 minutes on a rotator, after which 5 μg of SMNP was added and incubated with antigen-alum formulations for 30 minutes before immunization. This dose of SMNP corresponds to 5 μg of Quil-A saponin and 0.5 μg MPLA. Mice were immunized subcutaneously at the tail base with 50 μL on each side of the tail base with one of three formulations: alum-pSer, SMNP, or alum-pSer/SMNP.

### scRNA-seq study design, processing, and analysis

The inguinal dLNs of vaccinated BALB/c mice (n=14 animals for SMNP and alum-pSer/SMNP, n=29 for alum-pSer) were isolated 14 days post-immunization. dLNs were mashed and passed through a 70 μm filter to obtain single-cell suspensions. Cells were stained for viability (Thermo Fisher Live/Dead Fixable Aqua, 1:1000 in PBS, 20 min at 4°C) and labeled with antibodies (1:200 in PBS+1%BSA, 30 min at 4°C) against CD3e (BV711, BioLegend, 145–2C11clone), CD19 (APC, BioLegend, 6D5 clone), B220 (PE-Cy7, BioLegend RA3–6B2 clone), CD38 (FITC, BioLegend 90 clone), GL7 (PerCP-Cy5.5, BioLegend GL7 clone), and TotalSeq-A cell hashing antibodies (BioLegend). Antigen-specific staining was completed using 2.4 μg biotinylated MD39 conjugated to 0.4 μg streptavidin-BV421 (BioLegend) and 0.4 μg streptavidin-PE (BioLegend) in 50 μL PBS+1%BSA at 4°C. Antigen double-positive GC B cells were sorted on a BD FACS Aria (BD Biosciences) cell sorter and processed immediately following the SeqWell protocol (table S2) (29, 30). scRNA-seq libraries were sequenced by Illumina NovaSeq and aligned to the GRCm39 reference genome using the STARsolo pipeline (version 2.4.0) (82). The gene expression count matrix and cell hashing sequence reads were processed and analyzed using Seurat v5.0.1, ggplot2 v3.5.1, CITE-seq-Count v1.4.2, Scanpy v1.10.4, and scVelo v0.3.3 (83–86).

Myc- and mTORC1-target genes (table S1) were retrieved from the literature (48, 52, 53) and their gene set module scores were calculated using the AddModuleScore() function in Seurat (83). Data from Mathew *et al*., King *et al*., and Holmes *et al.* were obtained from ArrayExpress with accession numbers E-MTAB-9478, E-MTAB-9005, and from Gene Expression Omnibus database GSE139891, respectively (44–46). Differentially expressed genes of relevant clusters from these studies were mapped onto our scRNA-seq data using the AddModuleScore() function (83).

### scBCR-seq library generation, sequencing, processing, and analysis

The scBCR-seq library was prepared following the B3E-seq protocol as described previously (31). In brief, the immunoglobulin (Ig) transcripts were enriched and amplified from the 3’-barcoded cDNA (table S3). A set of V-gene primers (modified from Tillers *et al*. (87)) (table S4), were used to further enrich immunoglobulin transcripts before sequencing (table S5 and S6). BCR sequence reads were processed with pRESTO (v0.5.13), Change-O (v0.4.6), UMI-Tools, and IgBlast (v1.14.0) (88–91) to reconstruct and annotate full-length BCR sequences that match corresponding single-cell transcriptomes. The NHP BCR-seq data from Phung *et al*. 2024 (57) was retrieved from Sequence Read Archive (SRA) with accession number PRJNA1016452 and processed as described by the authors.

Clonally related sequences were identified using the DefineClones.py function (Change-O v0.4.6) with an 85% CDR3 nucleotide similarity threshold determined by distToNearst() function from the Shazam package (89). Germline sequences were inferred using the CreateGermline.py function (Change-O v0.4.6) (89). Public clonotypes were identified based on the shared V gene, J gene, CDR3 junction length, and 90% CDR3 amino acid similarity threshold determined by distToNearst() function for both mouse and NHP datasets. Clone sizes, or the number of cells per clone, were calculated using the countClones() function (alakazam v1.2.1). Clonal evenness was quantified for each mouse by Pielou’s evenness index (*J*) (54). It was calculated as the Shannon Diversity Index (*H*) divided by the natural log of the total number of clones in each mouse (56). A *J* value of 1 means that all the clones in the mouse have the same number of cells.

SHM counts were aggregated using the observedMutations() (Shazam v1.1.2) function (89). The Circos plots were generated using the *circlize* package (92). BCR pairing diversity was calculated in the following steps: first, cells were collapsed into their respective clones. If a clone matched to more than one light chain V gene, the light chain V gene with the most cells in the clone was designated for the clone. Second, clones were aggregated into unique heavy and light-chain V gene pairs, and the number of clones using a V gene pair was counted. Lastly, Shannon’s Diversity Index was calculated for each group, whereby each unique V gene pair was considered the “species,” and the number of expanded clones using the pair was the “abundance” for the “species.” The same calculation was also performed at the individual mouse level.

### Confocal microscopy and quantification

Imaging was performed on a Leica SP8 confocal microscope using white light and argon lasers with spectral emission filters with a minimum bandwidth of 10 nm. A 25X water-immersion objective was used unless otherwise indicated. Laser and detector settings were kept constant across all imaging time points for each antigen. The fraction of FDC occupied by antigen was quantified as described previously (58, 61). Briefly, autofluorescence was first zeroed out from the signal channels by applying a high-pass filter to binarize and identify autofluorescence locations for each z-height slice in the imaged sample. Next, a sum intensity z-projection on the combined CD35^+^ (FDC) channels was binarized and used to define a selection comprising the FDC area. Finally, a sum intensity z-projection on the immunogen signal channel was binarized using a high pass filter. This binary mask was used to multiply a sum intensity z-projection such that all dim pixels were zeroed and all bright pixels retained their unaltered intensity information. Only the bright pixels in the immunogen signal channel were ratiometrically quantified against the FDC area to identify the fraction of FDC occupied by antigen. The antigen signal at the injection site was quantified by creating a binary mask of pixels containing antigen signals first, and the signal intensity was then calculated by summing the signals and dividing by the pixel area of the binary mask. FRET efficiency was calculated as previously described (61). Images were processed and analyzed in ImageJ (version 2.1.0/1.53c).

### Statistical analysis

Individual-level data are presented in data file S1. For sequencing analysis, statistical analysis was performed in the R software version 4.3.0. The specific statistical tests are indicated in the figure legends. All other data were plotted and all statistical analyses were performed using GraphPad Prism 9 software. All graphs display mean values, and the error bars represent the standard error of the mean (s.e.m.) unless otherwise specified. Two-sided testing was used unless otherwise specified. Multiple testing adjustments to alpha levels were indicated in the figure legends. Animals with fewer than 10 recovered B cells and 3 recovered BCR sequences were excluded from the statistical analyses; otherwise, no samples or animals were excluded. Statistical comparison was performed using a one-way ANOVA followed by Tukey’s post-hoc test for single timepoint data with three or more groups and two-way ANOVA followed by Tukey’s post-hoc test for multi-timepoint longitudinal data unless otherwise indicated. Data were considered statistically significant if the p-value was less than 0.05.

## Supplementary Material

Supplementary data file

MDAR Reproducibility Checklist

3

List of Supplementary Materials

Materials and Methods

Fig. S1 to S9

Tables S1 to S6

References (93) (94) (95)

MDAR Reproducibility Checklist

Data file S1

## Data Availability

All data associated with this study are in the paper or supplementary materials. The scRNA-seq and BCR-seq data generated in this study are available in the Gene Expression Omnibus (GEO) under accession number GSE288450. Codes for preprocessing and analysis of single-cell data are available on GitHub at https://github.com/ymjzhang/pSer-SMNP_STM_2025 and on Zenodo at https://doi.org/10.5281/zenodo.15208275 (96). SMNP adjuvant can be shared via MTA upon reasonable request.
