# Supplementary material for: Vaccines combining slow release and follicle targeting of antigens increase germinal center B cell diversity and clonal expansion
Source: Sci Transl Med. Author manuscript; Available in PMC 2025 Aug 10. (PMC12335825; doi:10.1126/scitranslmed.adw7499)
Supplement: 3 [file NIHMS2092992-supplement-3.pdf]

Supplementary Materials and Methods

Phosphoserine peptide synthesis

$pSer_4$-maleimide and Ser_4-maleimide peptide linkers were synthesized using solid phase synthesis on low-loading TentaGel Rink Amide resin (0.2 meq/g, Peptides International) as described previously *(18)*. Briefly, resin was deprotected with 20% piperidine (Sigma Aldrich) in dimethylformamide (DMF, Sigma Aldrich), and peptide couplings were performed with 4 equivalents of Fmoc-Ser(PO(OBzl)OH)-OH (Millipore Sigma) (or Fmoc-Ser(tBu)-OH for Ser linkers) and 3.95 equivalents of hexafluorophosphate azabenzotriazole tetramethyl uranium (HATU) for 2 hours at 25°C in dichloromethane and DMF (1:2 vol:vol). pSer residues were deprotected with 5% 1,8-diazabicyclo[5.4.0]undec-7-ene (DBU) in DMF for 45 min. Double couplings were performed for the third and fourth residues. An Fmoc-protected 6-unit oligoethylene glycol linker (Peptides International DPG-5750) was then coupled to the peptide, deprotected, and reacted with N-maleoyl-β-alanine (Sigma Aldrich). Completion of each deprotection and coupling step was confirmed by a ninhydrin test (Sigma Aldrich). pSer side chains were deprotected and the peptide was cleaved from the resin in 95% trifluoroacetic acid (Sigma Aldrich), 2.5% H_20, and 2.5% triisopropylsilane (Sigma Aldrich), for 2.5 hours at 25°C. The product was precipitated in 4°C diethyl ether (Sigma Aldrich) and dried under N_2, then purified by HPLC on a C18 column (Agilent Zorbax 300SB-C18) using 0.1 M triethylammonium acetate buffer (Glen Research) in an acetonitrile gradient. The peptide mass was confirmed by matrix-assisted laser desorption/ionization-time of flight mass spectrometry.

SMNP adjuvant synthesis

Saponin-MPLA nanoparticle (SMNP) adjuvant was prepared as previously described *(25)*. Briefly, solutions at 20 mg/mL were prepared of cholesterol, DPPC, and PHAD MPLA (Avanti Polar Lipids) in 20% MEGA-10 detergent (Sigma). Quil-A saponin (InvivoGen) was dissolved in Milli-Q water at a final concentration of 100 mg/mL. These stock solutions were mixed at a mass ratio of 10:2:1:1 Quil-A:chol:DPPC:MPLA and diluted in phosphate-buffered saline (PBS) to a final cholesterol concentration of 1 mg/mL. The solution was equilibrated overnight at 25°C and then dialyzed for 5 days against PBS (5 L PBS volume for 50mg Quil-A scale; PBS replaced every 12 hours) using a 10kDa MWCO cassette (Thermo Fisher) to remove detergent. The adjuvant was then sterile filtered and purified by FPLC using a Sephacryl S-500 HR size exclusion column (Cytiva Life Sciences). SMNP was concentrated to approximately 3 mg/mL relative to Quil-A using Amicon Ultra Centrifugal Filters (50 kDa MWCO, Millipore Sigma), and the final concentration of SMNP was determined using a cholesterol quantification assay (Sigma Aldrich).

Germinal center (GC) response analysis

Inguinal lymph nodes were collected from immunized mice at indicated time points. For germinal center analysis shown in Fig. 1B and C and fig. S2B, cells were stained for viability (Thermo Fisher Live/Dead Fixable Aqua, 1:1000 in PBS, 20 min at 4°C), labeled with antibodies (1:200 in PBS+1%BSA, 30 min at 4°C) against CD3e (BV711, BioLegend, 145-2C11 clone), B220 (PE-Cy7, BioLegend, RA3-6B2 clone), CD38 (FITC, BioLegend, 90 clone), and GL7 (PerCP-Cy5.5, BioLegend, GL7 clone), and antigen-specific staining was completed using 2.4

μg biotinylated MD39 conjugated to 0.4 μg streptavidin-BV421 (BioLegend) and 0.4 μg streptavidin-PE (BioLegend) in 50 μL PBS+1%BSA at 4°C. Samples were analyzed by flow cytometry on a BD Celesta and analyzed on FlowJo. For memory B cell analysis shown in Fig. 1F, cells were stained for viability (Thermo Fisher, Zombie NIR, 1:1000 in PBS, 20 min at 4°C), labeled with antibodies (1:200 in PBS+1%BSA, 30 min at 4°C) against CD3e (APC-Cy7, BioLegend, 145-2C11 clone), B220 (BUV496, BD, RA3-6B2 clone), IgD (PE, BioLegend, 11-26c.2a clone), CD73 (BV605, BD, TY/11.8 clone), PD-L2 (BUV395, BD, TY25 clone), and antigen-specific staining was completed using 2.4 μg biotinylated MD39 conjugated to 0.4 μg streptavidin-BB515 (BioLegend) and 0.4 μg streptavidin-APC (BioLegend) in 50 μL PBS+1%BSA at 4°C. For BrdU$^+$ and DZ/LZ GC B cell analysis shown in Fig. 2G to I, cells were stained for viability (Thermo Fisher, Zombie NIR, 1:1000 in PBS, 20 min at 4°C), labeled with antibodies (1:200 in PBS+1%BSA, 30 min at 4°C) against B220 (BUV496, BD, RA3-6B2 clone), CD38 (BUV737, BD, 90/CD38clone), GL7 (RB705, BD, GL7 clone), CD86 (PE, BioLegend, GL-1 clone), CXCR4 (BUV395, BD, 2B11/CXCR4 clone), and antigen-specific staining was completed using 1.2 μg biotinylated MD39 conjugated to 0.4 μg streptavidin-BV421 (BioLegend) in 50 μL PBS+1%BSA at 4°C. For WT versus *Cr2* KO GC B cell analysis shown in Fig 6C and D, cells were stained for viability (Thermo Fisher Fixable Far Red, 1:1000 in PBS, 20 min at 4°C), labeled with antibodies (1:200 in PBS+1%BSA, 30 min at 4°C) against CD45.2 (FITC, Thermo Fisher, 104 clone), CD45.1 (BV785, BD, A20 clone), CD90.2 (AF700, BioLegend, 30-H12 clone), GL7 (BV421, BD, GL7 clone), CD38 (PE-Cy5, Thermo Fisher, 90 clone), CD19 (PE-Cy7, BD, 6D5 clone), B220 (BV805, BD, RA3-6B2 clone), and antigen-specific staining was completed using 2.4 μg biotinylated MD39 conjugated to 0.4 μg streptavidin-PE (BioLegend) and 0.4 μg streptavidin-APC (BioLegend) in 50 μL PBS+1%BSA at 4°C. Samples were analyzed by flow cytometry on a BD Symphony A3 and analyzed on FlowJo.

Serum enzyme-linked immunosorbent assay (ELISA)

Serum was collected from mice retro-orbitally using capillary tubes and stored at -20°C until analysis. To determine serum IgG titers, Nunc Maxisorp plates (Invitrogen) were coated with Galanthus nivalis lectin (Sigma Aldrich) at 2 μg/mL for 4 hours at 25°C and blocked with 2% bovine serum albumin (BSA) in PBS for 16 hours at 4°C. Plates were subsequently washed with 0.05% Tween-20 in PBS and incubated with 2 μg/mL unmodified or pSer-conjugated MD39 in 2% BSA in PBS for 2 hours at 25°C. Serum dilutions (1:10 dilution followed by 1:50 dilution with 1:4 serial dilutions) were incubated in the plate for 2 hours. Plates are washed again, incubated with a goat anti-mouse IgG horseradish peroxidase (HRP)-conjugated secondary (BioRad) at 1:5000 dilution, and then developed with 3,3′,5,5′-tetramethytlbenzidine (Thermo Fisher), stopped with 2N sulfuric acid, and immediately read (450nm with 540nm reference) on a BioTek Synergy2 plate reader. Isotype ELISAs followed the same protocol but used goat anti-mouse IgG1 HRP cross-adsorbed secondary antibody, goat anti-mouse IgG2a HRP cross-adsorbed secondary antibody, or goat anti-mouse IgG2b cross-adsorbed secondary antibody (Invitrogen) at 1:2000 dilution.

scRNA-seq sequencing, alignment, processing, and analysis

cDNA was sequenced by Illumina NovaSeq 6000 SP. Sequencing reads were demultiplexed and aligned to the GRCm39 reference genome using the STARsolo pipeline (version 2.4.0) *(74)* on Terra.Bio with default parameters except star_version = "2.7.10b", soloType = "CB_UMI_Simple", soloCBstart = 1, soloCBlen = 12, soloUMIstart = 13, soloUMIlen = 8, soloCellFilter= "TopCells 10000" for Seq-Well-specific libraries. The gene expression count matrix was processed using the Seurat (v5.0.1) package in R. The initial quality control filtered out genes that were detected in less than 3 cells and removed cells with less than 300 genes or greater than 10% mitochondrial genes. Cell hashing sequence reads were aligned to hashtag oligo (HTO) barcodes using CITE-seq-Count v1.4.2. The HTO count matrix was added to the Seurat object and normalized. The HTODemux() function was used to assign HTO to each cell. Only singlet cells by HTO assignment were kept for downstream analysis.

Cells were normalized using the NormalizeData() function. The cell cycle was predicted using the CellCycleScoring() function, and variable genes were identified using the FindVariableFeatures() function. The ScaleData() function was used to regress out RNA feature counts and percent of mitochondrial genes before performing principal component analysis (PCA) using the RunPCA() function. Batch correction was performed using the Harmony method within the IntegrateLayers() function. Fifteen principal components (PCs) and 500 decision trees were used for constructing the nearest-neighbor graph with the FindNeighbors() function. Thirty neighboring points and twenty PCs were used to generate uniform manifold approximation and projection (UMAP) with the RunUMAP() function. Unsupervised clustering was determined using Louvain clustering as implemented in the FindClusters() function. Differential gene expression analysis was performed using FindMarkers() and FindAllMarkers() functions with the Wilcoxon Rank Sum test, and the p-values were adjusted using Bonferroni correction.

Exonic (spliced) and intronic (unspliced) transcripts were counted with STARsolo, integrated with scRNA-seq counts, and normalized using the Scanpy v1.10.4 package. The dynamical model from the scVelo v0.3.3 package was used to estimate RNA velocity, where thirty PCs and thirty neighbors were used to calculate the moments for velocity estimation. The estimated velocity was visualized as vector fields projected onto the UMAP embeddings. Velocity-based pseudo-temporal relationships among cells were approximated using the scvelo.tl.latent_time() function to infer the real-time experienced by cells.

Enrichment of immunoglobulin transcripts

The enrichment of immunoglobulin (Ig) transcripts from SeqWell 3'-barcoded whole transcript amplification (WTA) products was performed using xGen Lockdown reagents (IDT, cat. no. 1072281). Biotinylated probes for *Ighm, Ighd, Ighg1/2, Ighg3, Igha, Igkc, Iglc,* and *Iglc2/3* were synthesized by IDT and were used at a concentration of 1.5 µM (each; 7.5 µM for heavy chain and 4.5 µM for light chain in total) (**table S3**). We performed the enrichment for heavy chains and light chains separately. 3.5 µL of SeqWell WTA product was combined with 8.5 µL 2x hybridization buffer, 2.7 µL hybridization buffer enhancer, 0.8 µL Seq-Well WTA primer (40 µM), 1uL probe mix, and 0.5 µL mouse cot-1 DNA (Invitrogen, cat. No. 18440-016) were combined and let sit at room temperature for 5 min. The mixture was then incubated at 95°C for 10 min and 65°C for one hour. The pull-down steps follow exactly according to the remainder of the xGen lockdown protocol. 50 µL of Dynabeads M-270 Streptavidin (Invitrogen, cat. no.

65306) were used for each sample. At the end of this protocol, Ig-bound beads were mixed in 20 µL of water.

The enriched products were then amplified with polymerase chain reaction (PCR). Five PCR reactions for each enriched sample were performed with the following composition per reaction: 12.5 µL of 2x Kapa Hifi Hotstart Readymix (Roche, cat. no. KK2602), 8.5 µL water, 2.0 µL SeqWell WTA primer (10 µM), and 2.0 µL of Ig-bound beads (**tables S2 and S3**). The following PCR cycling conditions were used: 1 cycle of 95°C, for 3 min; 25 cycles of 98°C for 40s, 67°C for 20s, 72°C for 1 min; 1 cycle of 72°C for 5 min. The reactions were then pooled and purified using a homemade SPRI reagent at a ratio of 0.80x for the light chain and 0.65x for the heavy chain.

scBCR-seq library generation and sequencing

We designed Nextera-IGHV, Nextera-IGKV, and Nextera-IGLV primer sets for mice based on Tiller *et al. (87)* with modifications (**table S4**). Reaction mixtures for heavy and light chains were composed of 12.5 µL 2x Kapa Hifi Hotstart PCR Readymix, 6.0 µL water, 2.5 µL primer mix (equimolar pooling of primers with a final concentration of 10 µM), and 4.0 µL of enriched product. Primer extension was performed with the following thermal program: 98°C, 5 min, 55°C, 30s, 72°C, 2 min for light chains; 98°C, 5 min, 60°C, 30s, 72°C, 2 min for heavy chains. The products were cleaned with SPRI at a ratio of 0.8x (light chain) or 0.65x (heavy chain) and were eluted into 12 uL of water.

Four reactions of library index PCR were performed per sample. Reactions were composed of 0.5 µL P5_index_TSO primer (10 µM), 0.5 µL P7_index_Nextera primer (10 µM), 12.5 µL 2x Kapa Hifi Hotstart Readymix, 9 µL of water, and 2.5 µL of primer extension product (**table S5**). Amplification used the following cycling conditions: 1 cycle, 95°C, 2 min; 14-20 cycles of 95°C, 30s, 60°C, 30s, 72°C, 1.5 min; 1 cycle of 72°C, 5 min. Reactions were pooled and purified using SPRI at a ratio of 0.8x for light chains and 0.65x for heavy chains.

Final products were assessed using an Agilent Tapestation and D5000 ScreenTape (Agilent, cat. #5067-5588). Light chain libraries usually show a clean peak around 1000bp, and heavy chain libraries usually show a clean peak around 1500bp. Libraries were sequenced on an Illumina MiSeq using the 600-cycle kits or on an Illumina NovaSeq SP using the 500-cycle kits (**Table S5**). First, the Seq-Well R1 primer was used to sequence the cell barcode and UMI (20 nt on MiSeq, 26 nt on NovaSeq). Then, custom sequencing primers specific for the BCR constant region were used to sequence the BCR using the index 1 read (300 nt on MiSeq, 252 nt on NovaSeq). The 8-nucleotide i5 index barcode was sequenced using the Seq-Well R2 index primer (NovaSeq) or no custom primer (MiSeq). Lastly, the Nextera R2 primer was used to sequence the remainder of the BCR with read 2 (300 nt on MiSeq, 252 nt on NovaSeq).

scBCR-seq library processing

The molecular identity (Read 1) and paired-end reads (Index Read 1 and Read 2) were assembled in silico with pRESTO (v0.5.13) and Change-O (v0.4.6) pipelines to reconstruct full-length BCR sequences that match corresponding single-cell transcriptomes. Reads with an average quality (Q) score below 21 were removed using the FilterSeq.py function. Cell barcode and UMI reads (Read 1) with a Hamming distance of up to one nucleotide mismatch were collapsed to correct potential sequencing errors using UMI-Tools. The MaskPrimers.py function

was used to annotate and filter reads with the correct isotype (Index Read 1) and V-region primer (Read 2); the corresponding primer sequences on the reads were masked as well. Only read pairs (Index Read 1 and Read 2) that passed both the FilterSeq.py and MaskPrimers.py processing steps were retained using the PairSeq.py function.

To alleviate library preparation-related technical errors or sequencing-related errors such as barcode swapping and amplicon truncation, the ClusterSeqs.py function was used to sub-cluster Index Read 1 (3'-isotype) and Read 2 (5'-V(D)J) reads separately for each unique Ig molecule (cell barcode + UMI). The cluster fraction was calculated as the number of reads in each sub-cluster divided by the total number of detected reads for the Ig molecule (cell barcode + UMI). Only the cluster of reads with a cluster fraction > 0.5 was kept. Next, the BuildConsensus.py function was used to collapse and call consensus sequences on Index Read 1 and Read 2 separately for each unique Ig molecule (cell barcode and UMI). The AssemblePairs.py function was used to assemble these paired-end consensus sequences into a single overlapping sequence. The resulting sequences were annotated with the AssignGenes.py function and then analyzed with IgBlast (v1.14.0) using reference sequences provided by IMGT.

Ig molecular consensus sequences with fewer than three reads and more than four ambiguous "N" characters were discarded. BCR sequences were matched to single cells using the 12 bp single-cell barcodes. To define a consensus BCR sequence for each cell, we performed single-linkage clustering on the recovered IMGT-gapped sequences using Levenstein distance for each cell and clustered sequences less than five distances away. We extrapolate the cellular consensus sequence from the largest cluster by a recursive string consensus-building algorithm. If the resulting consensus retains ambiguous ("N") characters, we discarded the sequence with the fewest number of reads in the cluster and re-attempted to determine a consensus sequence until no ambiguous character remained. This process allowed us to combine information from multiple BCR molecules recovered from the same cell into a single cell-level consensus sequence.

Electron microscopy

100 µg of BG505 SOSIP, recombinantly expressed and purified by 2G12 affinity and size-exclusion chromatography as described previously *(93)*, was incubated with 375 µg SMNP for 1 hour at room temperature. After 1 hour, the mixture was diluted using Tris-buffered saline to approximately 0.03 mg/mL BG505 SOSIP concentration and a 3 µL drop was applied to carbon-coated copper mesh grids, blotted, and stained for 60 s with 2% (w/v) uranyl formate. Automated data collection was set up using Leginon *(94)* on a 120 keV FEI Tecnai TF20 with a TVIPS TemCam F416 CMOS camera (62,000X magnification resulting in a 1.677 Å pixel size). Micrographs were saved and viewed in the Appion database *(95)*.

Antigen labeling

Cy3- and Cy5-labeled proteins were prepared as previously described *(61)* by diluting 1 mg/mL antigen solutions in PBS at 1:1 (v/v) in 0.2 M sodium bicarbonate buffer (Sigma Aldrich, pH 8.4). Stock solutions of Sulfo-Cyanine 3 and Sulfo-Cyanine 5 NHS esters (Lumiprobe) were prepared fresh in 0.2 M sodium bicarbonate pH 8.4 and added to the antigen. After reacting for 16 hours at 4°C, the solutions were desalted using Zeba Spin Desalting columns (40kDa

MWCO, Thermo Fisher), equilibrated in PBS, and 0.22 µm sterile filtered (Millipore Sigma). Antigens were stored at 4°C. The degree of labeling was determined by UV-vis spectroscopy using the extinction coefficients of the Sulfo-Cy3 and Sulfo-Cy5 NHS ester dyes, 162000 and 271000 M^{-1} cm^{-1}, respectively.

Antigen-alum binding and release

Alexa dye-labeled antigen was mixed with Alhydrogel (alum, InvivoGen) in tris-buffered saline at a 1:10 antigen:alum mass ratio at an alum concentration of 0.1 mg/mL, unless otherwise specified, for 30 min on a tube rotator at 25°C. To assess antigen binding to alum, samples were immediately centrifuged at 10,000x*g* for 10 min to pellet alum, and the fluorescence of the supernatant was measured against a standard curve of labeled antigen. To assess the release of antigen from alum, mouse serum was added to antigen-alum solutions post-loading to a final mouse serum concentration of 10 vol% and incubated at 37°C for 24 hours, unless otherwise specified. Samples were subsequently centrifuged at 10,000x*g* for 10 min to pellet alum, and the fraction of protein bound to alum was measured by fluorescence analysis of the supernatant using a Tecan Infinite M200 Pro plate reader.

Antigenicity profiling of immunogens

Antigenicity profiling of antigens was carried out by coating free trimer or alum-bound trimer on ELISA plates and assessing binding of serial dilutions of selected structure-sensitive monoclonal antibodies to the immunogens by ELISA. To capture alum on Nunc Maxisorp ELISA plates (Invitrogen), plates were first coated with $pSer_4$-conjugated cytochrome C at 2 µg/mL in PBS for 4 hours at 25°C. Alum was then added at 200 µg/mL in PBS to be captured by $pSer_4$-cytochrome C overnight at 4°C. To capture "free" MD39, plates were coated with mouse VRC01 antibody, rabbit 12N antibody, or Galanthus nivalis lectin (Sigma Aldrich) at 2 µg/mL for 4 hours at 25°C and blocked with 2% BSA in PBS for 16 hours at 4°C. For both alum-coated and antibody-coated plates, plates were subsequently washed with 0.05% Tween-20 in PBS and incubated with 2 µg/mL unmodified or pSer-conjugated MD39 in 2% BSA in PBS for 2 hours at 25°C. Indicated monoclonal antibodies were added to antigen-coated plates at 5 µg/mL with 1:4 serial dilutions for 2 hours at 25°C. Plates were washed and antibody binding was detected with a goat anti-human HRP conjugated secondary antibody with minimal cross-reactivity (Jackson ImmunoResearch) at 1:5000 dilution in PBS containing 2% BSA and then developed with 3,3′,5,5′-tetramethylbenzidine (Thermo Fisher), stopped with 2N sulfuric acid and immediately read (450nm with 540nm reference) on a BioTek Synergy2 plate reader. For antigenicity profiling after digestion with trypsin, plates were coated with rabbit 12N antibody and antigens were captured as described, followed by incubation with 0.1 mg/mL of TPCK-treated trypsin (Thermo Fisher) at 37°C for 2 hours prior to antigenicity profiling ELISA.

In vitro trypsin digestion of antigen

Stability of FRET dye-labeled MD39 trimer was assessed in vitro using TPCK-treated trypsin (Thermo Fisher). Trypsin was reconstituted at 2 mg/mL in 0.1 M ammonium bicarbonate buffer (Sigma Aldrich) pH 8 and subsequently mixed 1:1 (v/v) with 40 µg/mL of antigen in 0.1 M ammonium bicarbonate buffer pH 8. Samples were incubated at 37°C on a tube rotator for 2 to

16 hours. Antigens were subsequently coated on glass coverslips at 10 µg/mL for 16 hours at 4°C and washed in PBS prior to FRET imaging.

Tissue sectioning and staining

Inguinal lymph nodes were collected from immunized mice at varying timepoints post-immunization and placed into cryomolds containing optimal cutting temperature compound (Fisher Scientific) as previously described *(61)*. The injection sites were excised after shaving hair, including the subcutaneous fat with the injection site but excluding the muscle, and placed in cryomolds. The cryomolds were subsequently flash-frozen in 2-methylbutane (Millipore Sigma) pre-chilled in liquid nitrogen. Tissues were subsequently cryo-sectioned on a Leica CM1950 at 10 µm thickness and adhered to Superfrost Plus microscope slides (Fisher Scientific). Sections were stored at -80°C until use, at which point they were quickly thawed, fixed in 10% neutral buffered formalin for 8.5 min at 25°C, and washed for 10 min in PBS three times. Slides were subsequently incubated in blocking buffer (2% BSA with 0.1% Triton X-100 in PBS) for 30 min at 25°C and then stained. Injection sites were stained with CellMask Green (Thermo Fisher) at 1:5000 for 25 min at 25°C, washed for 10 min in PBS three times followed by Hoechst at 1:10000 (Thermo Fisher) for 10 min at 25°C. Lymph nodes were stained with BV421 anti-CD35 (BD Biosciences) at a 1:75 dilution for 2 hours at 25°C in blocking buffer. Slides were then washed for 10 min in PBS three times. PBS (9 µl) was added directly to the tissues before a #1.5 18 x 18 mm coverslip was placed over the sample and sealed with CoverGrip sealant (Biotium). Slides were stored at 4°C for a maximum of 1 week for imaging.

Cleared lymph node imaging

Inguinal draining lymph nodes were collected from mice on day 14 post-immunization and immediately placed in 1 mL 4% paraformaldehyde in a 24-well plate overnight at 4°C. The next day, the lymph nodes were washed twice in 1 mL PBS at room temperature for 30 min each, followed by soaking in 1 mL 50% PBS + 50% methanol for 30 min, then 1 mL 50% PBS + 50% methanol for 30 min, and in 1 mL 100% methanol. While the lymph nodes were in 100% methanol, a 3 mL bleach solution was prepared in a scintillation vial by mixing methanol, DMSO, and H_2O_2 in a 4:1:1 ratio. The lymph nodes were transferred to the bleach solution for 1 minute, transferred back to 100% methanol, and soaked for 30 min. After removing methanol, the lymph nodes were soaked in 1 mL 49.8% methanol + 49.8% tertiary butanol + 0.4% tocopherol for 30 min, then 1 mL 19.8% methanol + 79.8% % tertiary butanol + 0.4% tocopherol for 30 min, followed by 1 mL 99.6% tertiary butanol + 0.4% tocopherol at 37° C for one hour. After incubation, the lymph nodes were placed on a paper towel in a chemical fume hood to allow drying for about 5 min. Dried lymph nodes were transferred to glass scintillation vials containing 3 mL 99.6% dichloromethane + 0.4% tocopherol. The vials were swirled periodically for 3 to 6 min until the lymph nodes sank to the bottom of the vial, after which the lymph nodes were immediately transferred to scintillation vials containing 99.6% dibenzyl ether and 0.4% tocopherol. The lymph nodes were incubated at room temperature in the dark for 2 days before imaging on the Olympus FV1200 Laser Scanning Confocal Microscope.

Antigen-specific ELISpot assay

Bone marrow enzyme-linked immunospots (ELISpots) were performed in mice 5 weeks post-immunization following the manufacturer's protocol (MabTech, catalog no. 3825-2A) unless otherwise specified. Briefly, 96-well polyvinylidene difluoride ELISpot plates (Millipore Sigma, catalog no. MSIPS4510) were treated with 35% ethanol before coating with anti-mouse IgG at 15 μg/ml in sterile PBS overnight at 4°C. Cells were isolated from the femur and tibia of mice, ACK lysed, and 70-μm filtered in complete media (RPMI-1640 containing 10% fetal bovine serum, penicillin-streptomycin (100 U/ml), and 1 mM sodium pyruvate). The next day, the plate was blocked with complete media for at least 30 min before adding cells with three technical replicates per mouse. 400,000 cells were added per well, respectively, and incubated at 37°C with 5% CO_2 for 16 hours. The plate was then washed with PBS. Antigen-specific responses were determined by adding biotinylated MD39 (1 μg/ml) in PBS with 0.5% BSA to each well for 2 hours at 25°C. The plate was washed again in PBS and incubated with 1:1000 streptavidin–alkaline phosphatase in PBS with 0.5% BSA for 1 hour at 25°C. After washing, the plate was developed with bromochloroindolyl phosphate–nitro blue tetrazolium substrate (MabTech, catalog no. 3650-10) for 20 min, quenched with H_2O, and dried before quantification on an ImmunoSpot CTL analyzer.

Supplementary Figures

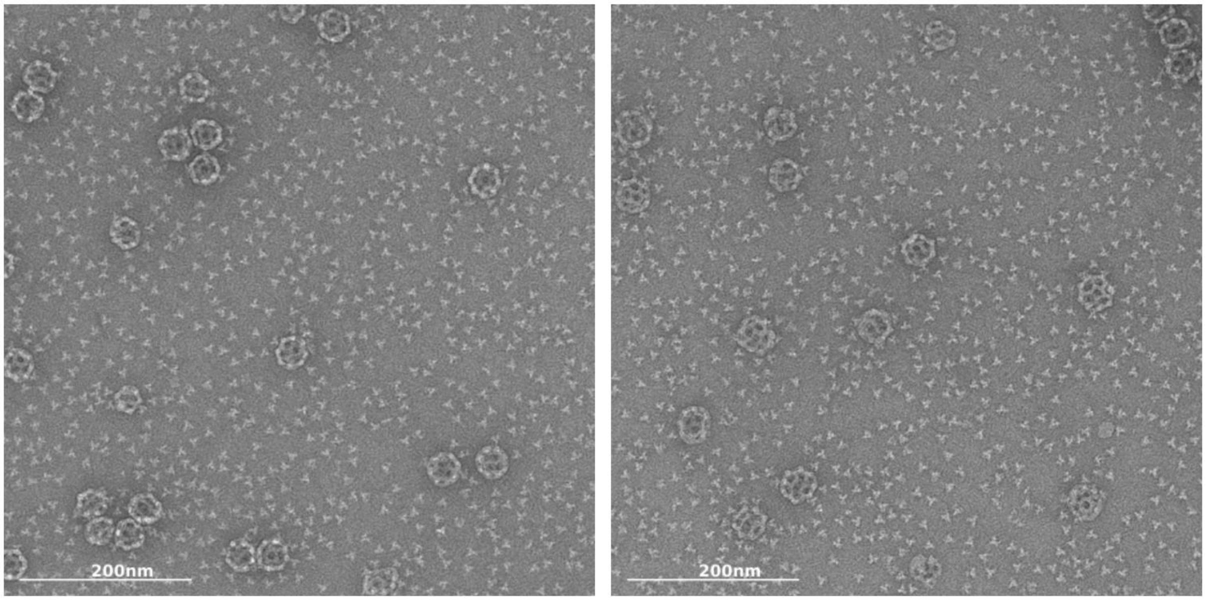

Fig. S1. SMNP does not interact with HIV Env trimer. Representative micrographs of 375 µg SMNP mixed with 100 µg BG505 SOSIP. Stained (with UF) and imaged after 1 hour incubation at 25°C. Scale bars represent 200 nm.

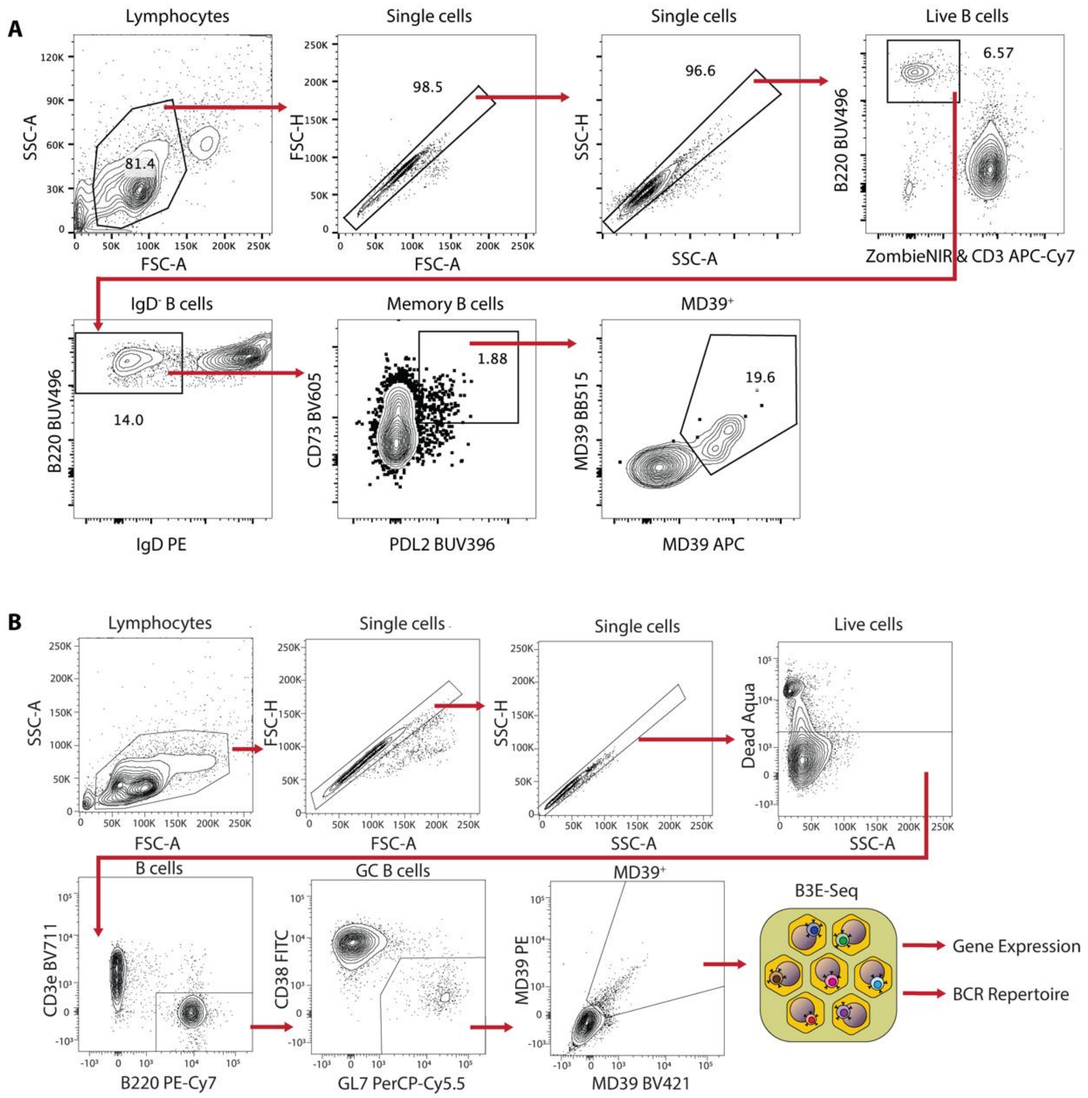

Fig. S2. Flow cytometry analysis for memory B cells and MD39^{+} GC B cells. (**A**) Representative flow cytometry gating strategy for MD39^{+} memory B cells. (**B**) The GC responses in draining lymph nodes were analyzed by flow cytometry, and MD39-binding GC B cells were sorted for scRNA-seq and scBCR-seq using SeqWell and B3E-seq protocols.

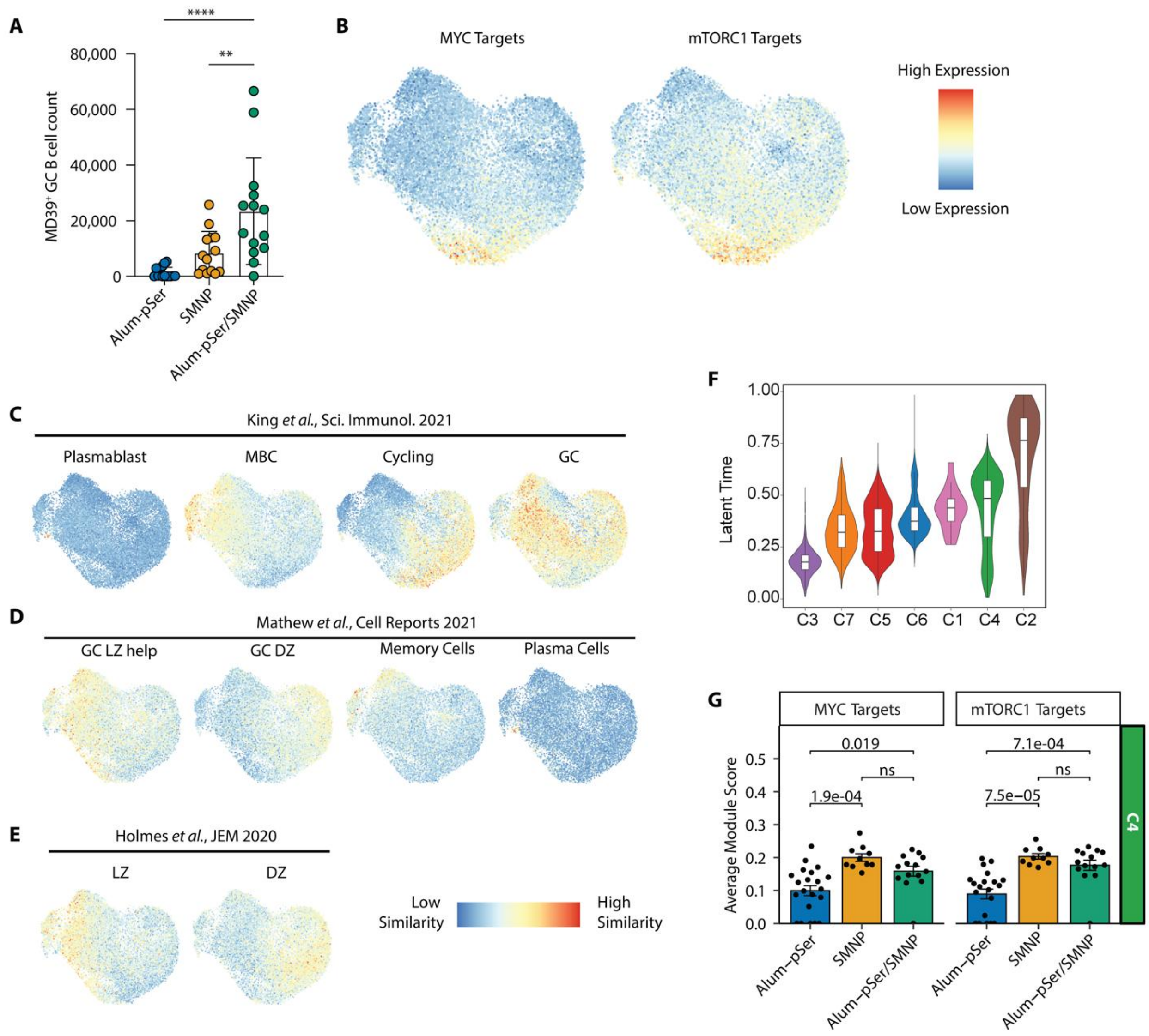

Fig. S3. scRNA-seq profiling of MD39-binding GC B cells. (**A**) BALB/c mice (*n*=14, 14, 14 for alum-pSer, SMNP, and alum-pSer/SMNP, respectively) were immunized with 5 μg MD39 Env trimer ± 50 μg alum ± 5 μg SMNP. GC B cell counts were detected by flow cytometry and loaded onto SeqWell arrays. Shown are mean ± s.e.m. Statistical significance was determined by one-way ANOVA followed by Tukey's multiple comparisons test. ** $p<0.01$, **** $p<0.0001$. For (B-G), analyses were performed using the same dataset as Fig. 2. (**B**) Uniform manifold approximation and projection (UMAP) of module scores of MYC- and mTORC1-targeted genes. High module scores suggest high expression of the gene set and are illustrated in red. Medium and low scores are illustrated in yellow and blue, respectively. The same color scheme is used for (C to E). (**C**) UMAP projection of gene expression signatures of plasmablast, memory B cell (MBC), cycling cells, and GC B cells defined among human tonsillar B cells by King *et al.* (**D**) UMAP projection of gene expression signatures of light zone (LZ), dark zone (DZ), memory B cells, and plasma cells defined among murine HA^+ B cells post LCMV infection by Mathew *et al.* (**E**) UMAP projection of gene expression signatures of LZ and DZ GC B cells defined among human tonsillar B cells by Holmes *et al.* (**F**) Violin plot of latent time of each recovered cell by

phenotypic clusters. (**G**) Average module score of MYC- and mTORC1-target genes among C4 cells per mouse by vaccine group. Statistical significance in (G) was determined by Kruskal-Wallis analysis of variance followed by Dunn’s post hoc test. ns indicates $p>0.05$.

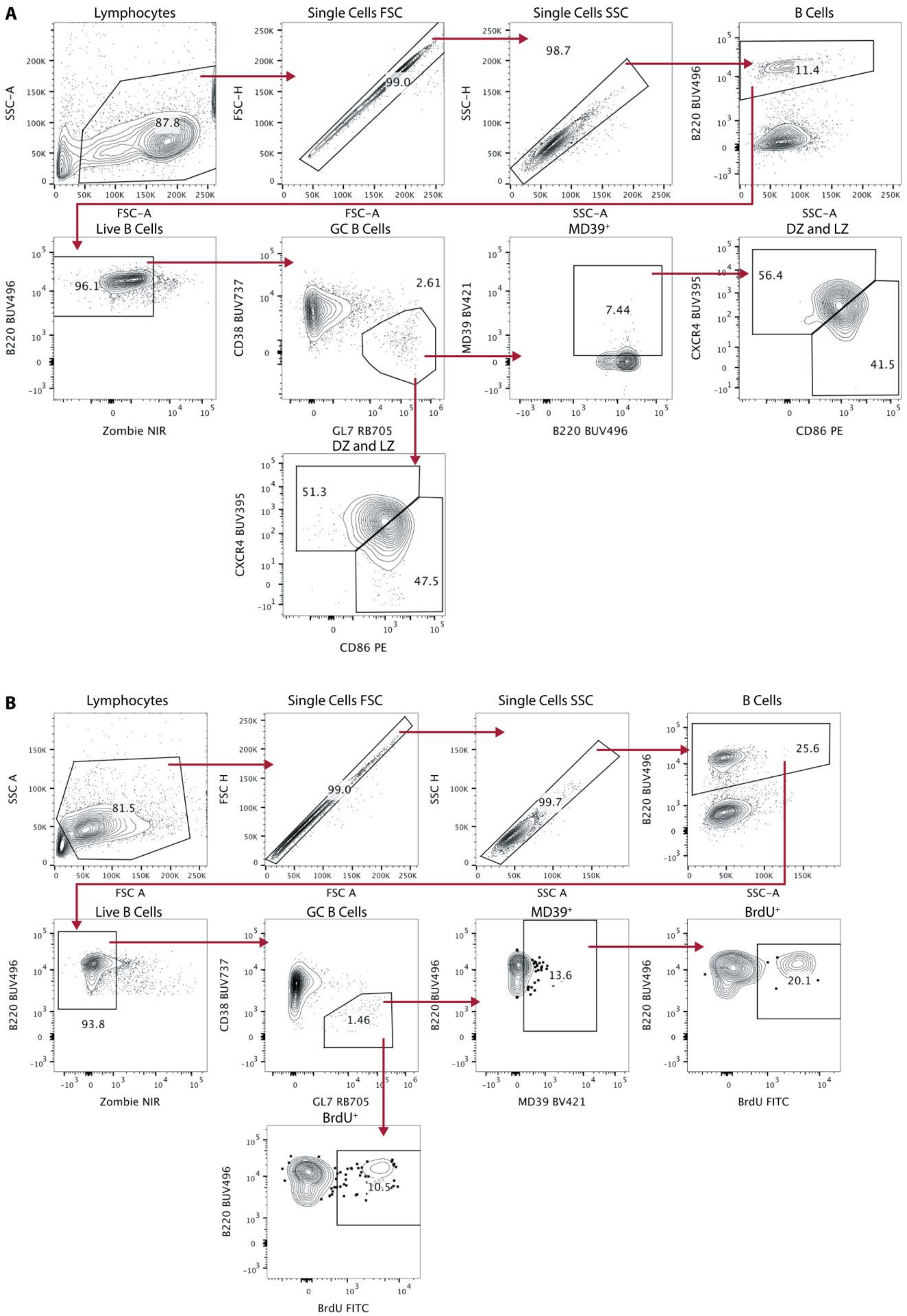
A
Lymphocytes
Single Cells FSC
Single Cells SSC
B Cells
87.8
99.0
98.7
11.4
SSC-A
FSC-H
SSC-H
B220 BUV496
FSC-A
FSC-A
SSC-A
SSC-A
Live B Cells
GC B Cells
MD39+
DZ and LZ
96.1
2.61
7.44
56.4
41.5
B220 BUV496
CD38 BUV737
MD39 BV421
CXCR4 BUV395
Zombie NIR
GL7 RB705
B220 BUV496
CD86 PE
DZ and LZ
51.3
47.5
CXCR4 BUV395
CD86 PE
B
Lymphocytes
Single Cells FSC
Single Cells SSC
B Cells
81.5
99.0
99.7
25.6
SSC A
FSC H
SSC H
B220 BUV496
FSC A
FSC A
SSC A
SSC-A
Live B Cells
GC B Cells
MD39+
BrdU+
93.8
1.46
13.6
20.1
B220 BUV496
CD38 BUV737
B220 BUV496
B220 BUV496
Zombie NIR
GL7 RB705
MD39 BV421
BrdU FITC
BrdU+
10.5
B220 BUV496
BrdU FITC

Fig. S4. Flow cytometry analysis of MD39-binding GC B cells. (**A**) Gating strategy for evaluating BrdU incorporation among total GC B cells and MD39-binding GC B cells. (**B**) Gating strategy for evaluating LZ ($CD86^+$) and DZ ($CXCR4^+$) total GC B cells and MD39-binding GC B cells.

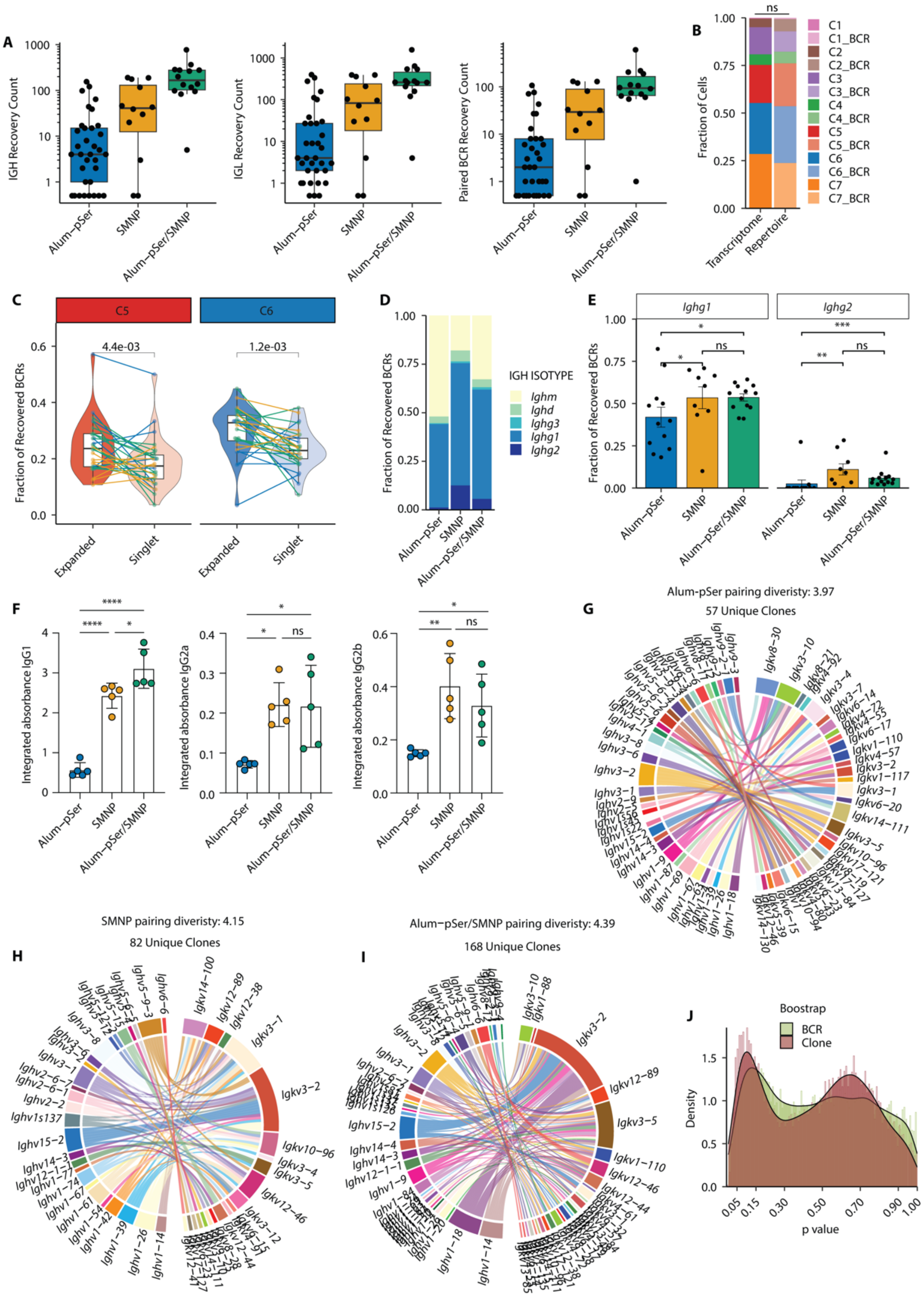

Fig. S5. BCR repertoire of MD39-binding GC B cells. Analysis is performed using the same dataset as Fig. 2. (**A**) Boxplot of the number of heavy chain, light chain, and paired BCR

sequences recovered from each animal by immunization group. (**B**) Cluster distribution of cells with paired BCR sequences recovered and all recovered cells. The p-value was computed by Chi-squared tests. (**C**) Violin and box plots of the fraction of cells from expanded versus singlet clones that were found in C5 and C6 per mouse. Only mice with both expanded and singlet clones recovered for the clusters were included in the analysis. The colored lines represent individual mice, connecting expanded clones and singlet clones found in the mouse. The alum-pSer group is illustrated in blue, SMNP in orange, and alum-pSer/SMNP in green. P values were computed using paired Mann-Whitney U test. (**D**) Distribution of heavy chain isotypes among MD39-binding GC B cells. (**E**) The fractions of *Ighg1* and *Ighg2* BCRs per mouse. Error bars are plotted as the s.e.m. The statistical significance was determined by Kruskal-Wallis analysis of variance followed by Dunn's post hoc test. (**F**) BALB/c mice (n=5 per group) were immunized with 5 µg MD39 ± 50 µg alum ± 5 µg SMNP. Serum IgG1, IgG2a, and IgG2b antibody responses were assessed at day 28 by ELISA using MD39 captured by lectin. Values plotted are the ELISA area under the curve (AUC) mean ± s.d. Statistical significance was determined by one-way ANOVA followed by Tukey's multiple comparisons test. ns $p>0.05$, * $p<0.05$, ** $p<0.01$, **** $p<0.0001$. (**G to I**) Chord diagrams illustrating the heavy and light chain pairings of expanded clones from mice vaccinated with formulations containing alum-pSer (G), SMNP (H), or alum-pSer/SMNP (I). Each chord on the diagram represents one clone, and the chord color represents the heavy chain V gene. Pairing diversity was calculated by the Shannon diversity index. (**J**) 200 BCRs or 100 clones were randomly sampled from the alum-pSer/SMNP and SMNP groups, pairing diversity scores were calculated for each mouse, and the p-value was computed with a two-tailed Wilcoxon test. This process was repeated 10,000 times to generate distributions of p-values.

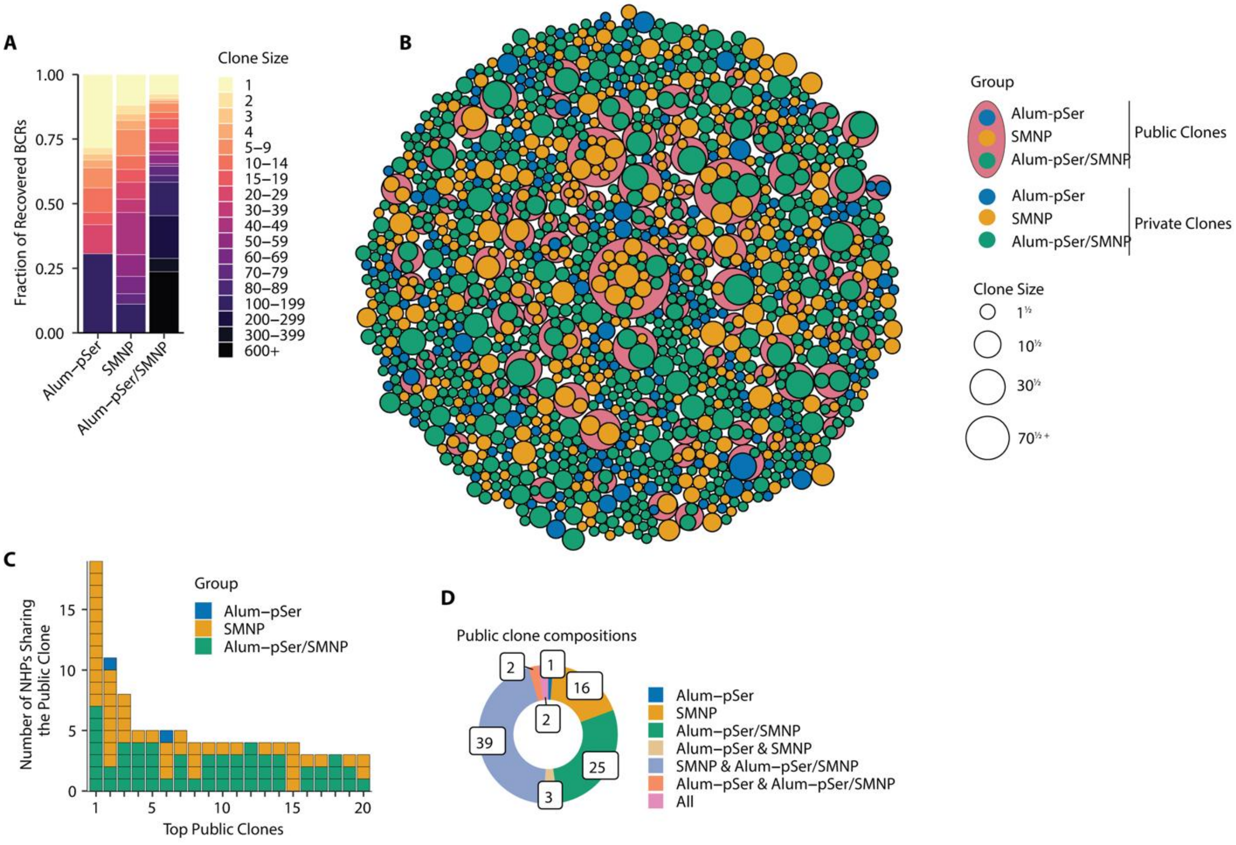

Fig. S6. Repertoire analysis of MD39-specific vaccine response in non-human primates (NHPs). The BCR sequencing data was retrieved from the Sequence Read Archive (SRA) with accession code PRJNA1016452. Only the relevant vaccine adjuvant groups and week 6 data were used for the re-analysis ($n_{\text{alum-pSer}} = 4$, $n_{\text{SMNP}} = 4$, $n_{\text{alum-pSer/SMNP}} = 5$). (**A**) Clone size distributions in each vaccine group. (**B**) Circle-packing diagram showing all recovered public and private clones. Singlet private clones are illustrated as individually colored circles (blue for the alum-pSer group, orange for SMNP, and green for alum-pSer/SMNP). Public clones are illustrated as coral-colored circles that enclose some private clones. The size of each private clone circle is proportional to the square root of its clone size. (**C**) The top 20 public clones by the number of animals sharing the public clone. (**D**) Donut plot showing the proportion and count of different compositions of public clones.

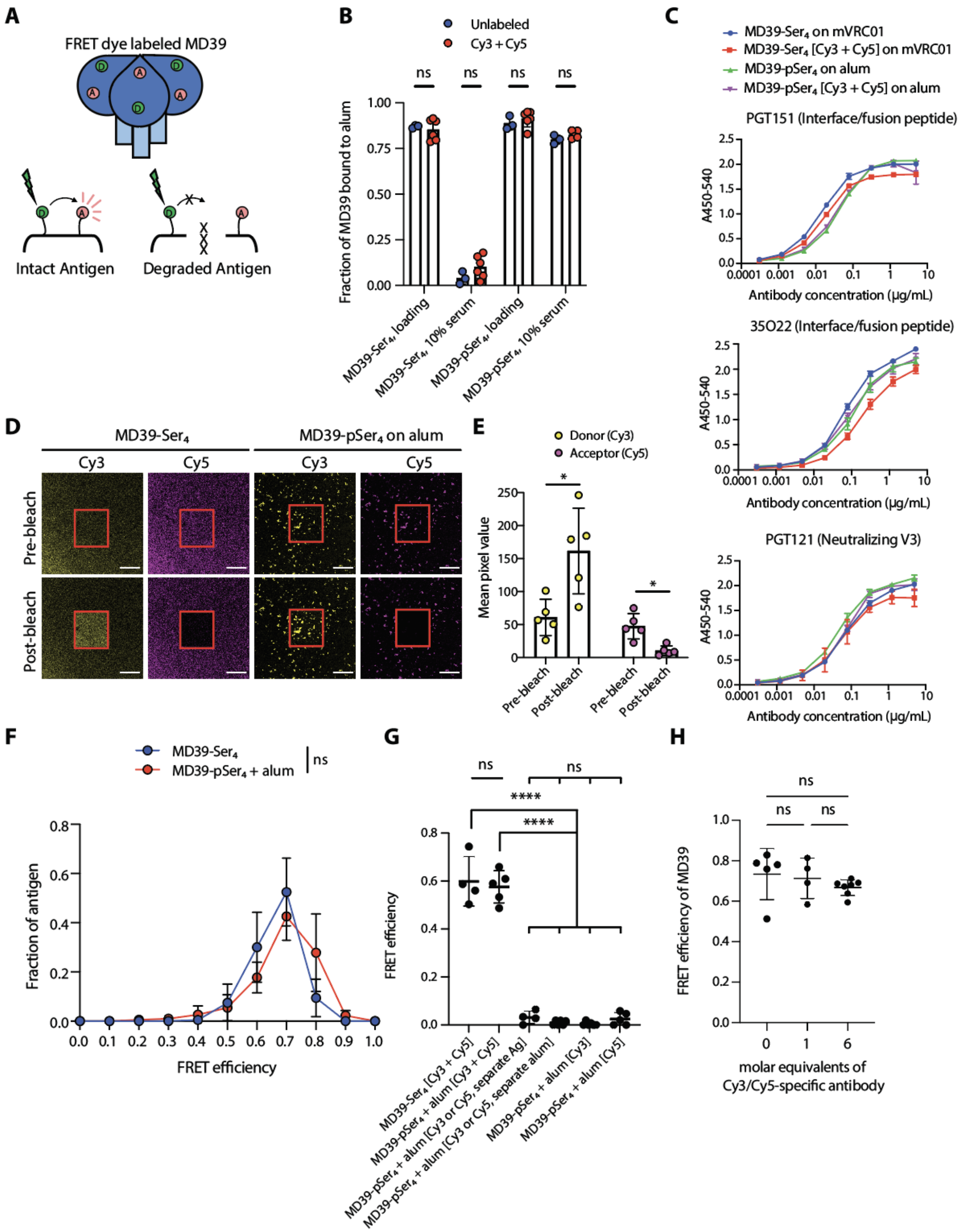

Fig. S7. FRET-based approach allows for the assessment of antigen stability. (**A**) Overview of FRET-based approach to investigate MD39 antigen stability. MD39 is labeled with donor and acceptor FRET pair dyes Cy3 and Cy5, such that FRET occurs when antigen is intact, and FRET does not occur when antigen is degraded. (**B**) Unlabeled (*n*=3) and C3/Cy5 labeled (*n*=6) pSer-

and Ser-conjugated MD39 trimers were mixed with alum, and the fraction of protein bound to alum after loading and after 24-hour incubation in 10% mouse serum at 37°C was assessed. Statistical significance was determined by two-way ANOVA followed by Sidak's multiple comparisons test. (**C**) Antigenicity profiling of unlabeled and Cy3 + Cy5-labeled MD39 trimers (*n*=3 replicates). MD39-pSer$_4$ was captured by alum and MD39-Ser$_4$ was captured by mVRC01. Values plotted are means ± s.d. (**D and E**) Acceptor (Cy5) photobleaching approach for soluble and alum anchored MD39 coated on glass coverslips (*n*=5 replicates). Acceptor photobleaching of intact antigen labeled with Cy3 and Cy5 results in a reduction in Cy5 emission and an increase in Cy3 emission (D), quantified in (E) for soluble MD39. Scale bar represents 50 µm. Red outline indicates region of photobleaching. Statistical significance was determined by paired one-way ANOVA followed by Tukey's multiple comparisons test. (**F**) Histogram of FRET efficiencies (*n*=4 to 5 replicates). Values plotted are means ± s.d. Statistical significance was determined by unpaired Student's t-test. (**G**) FRET efficiencies of indicated proteins coated on glass coverslips (*n*=4 to 5 replicates). Statistical significance was determined by one-way ANOVA followed by Tukey's multiple comparisons test. (**H**) MD39 was mixed with the indicated molar equivalents of a Cy3/Cy5-specific antibody and coated on glass coverslips (n=4 to 7 replicates), and their FRET efficiency was determined. Statistical significance was determined by one-way ANOVA followed by Tukey's multiple comparisons test. ns $p>0.05$, * $p<0.05$, **** $p<0.0001$. For (B, C, E, F, G, and H), values plotted are shown as means ± s.d.

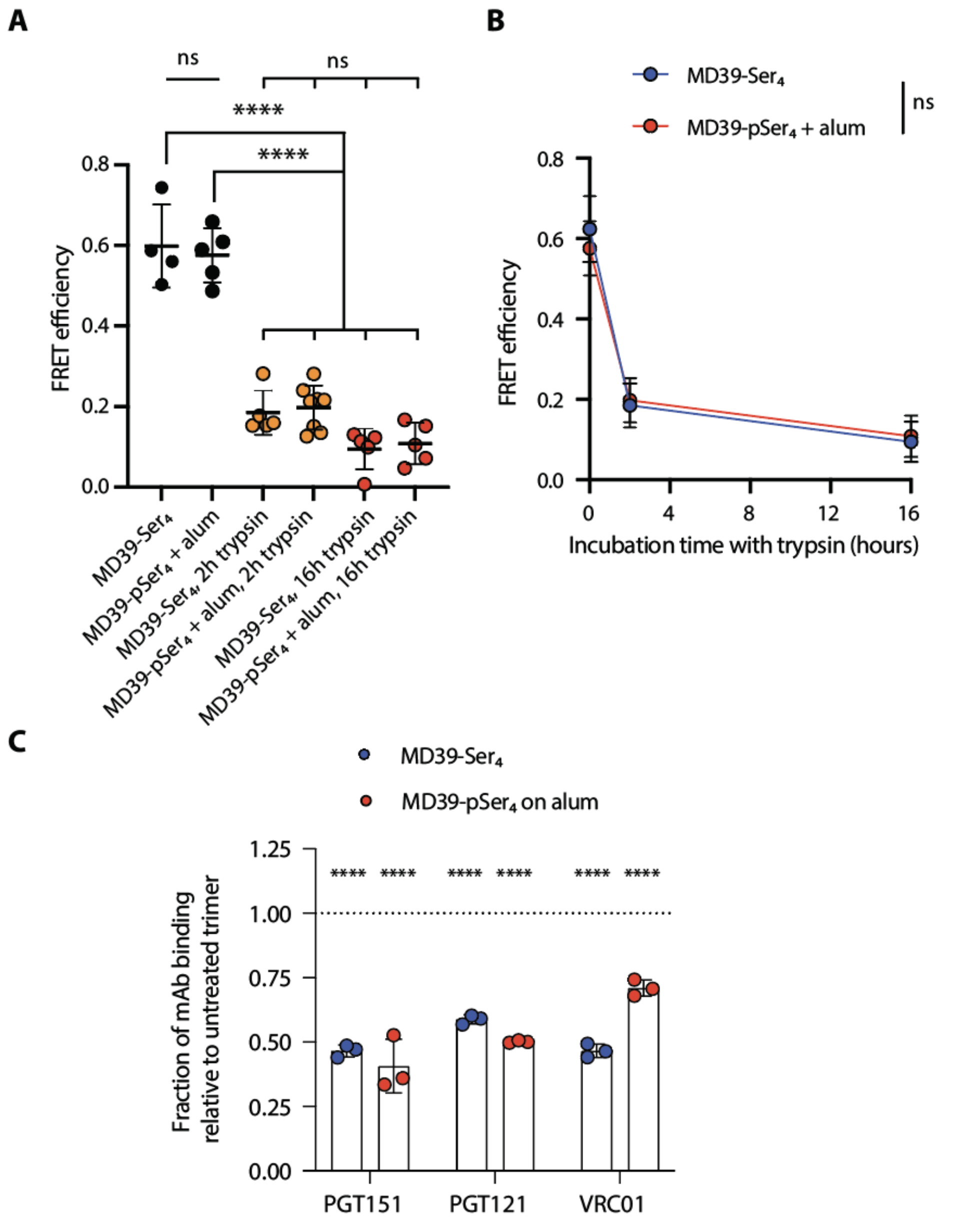

Fig. S8. FRET efficiency is correlated to MD39 stability. (**A and B**) FRET efficiencies of soluble MD39-Ser$_4$ and MD39-pSer$_4$ on alum (*n*=4 replicates), coated on glass coverslips following incubation with trypsin for 2 (*n*=8 replicates) or 16 hours (*n*=6 replicates) (A) or shown as longitudinal plot (B). (**C**) Antigenicity profiling of soluble MD39-Ser$_4$ captured by 12N antibody and MD39-pSer$_4$ captured on alum following incubation with trypsin for 2 hours at 37°C (*n*=3 replicates). Shown are integrated absorbance values following incubation with trypsin for 2 hours at 37°C normalized to signal not exposed to trypsin. Statistical significance was determined relative to the pre-2h incubation with trypsin values in (C). Values are plotted are means ± s.d. All data were analyzed by two-way ANOVA followed by Tukey's multiple comparisons test. ns p>0.05, **** p<0.0001. mAb, monoclonal antibody.

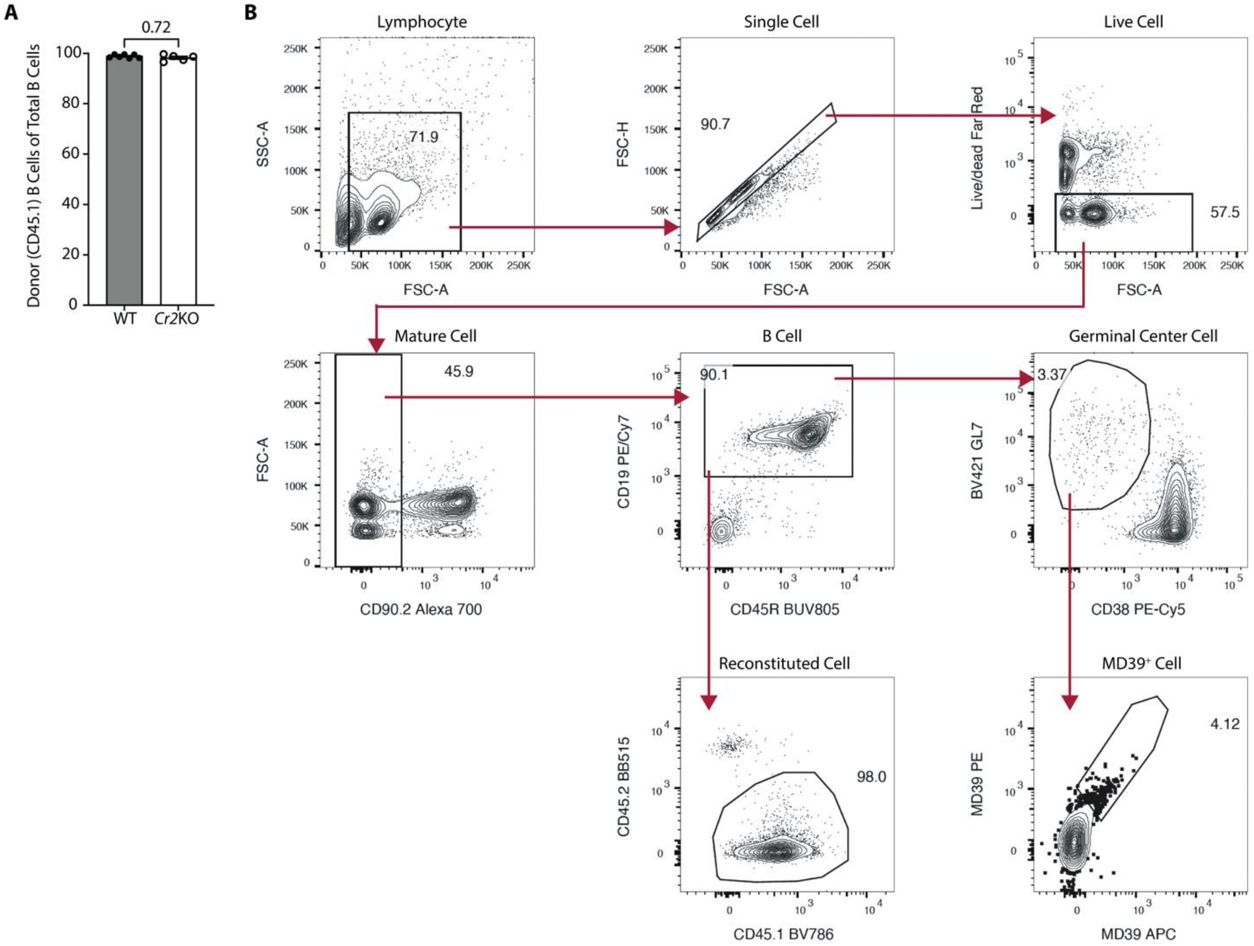

Fig. S9. Disrupting antigen accumulation on FDCs reduces antigen-specific GC responses. (A) Bar plot showing the percentages of CD45.1 B cells out of all B cells to confirm the success of reconstitution (n_{WT}=7, n_{Cr2KO}=5). The statistical significance was determined by two-tailed Mann-Whitney U tests. **(B)** Representative flow cytometry plots of MD39-binding GC B cells.

Table S1: Myc and mTORC1 target genes.

	**Target Genes**
**SCHUHMACHER_MYC_TARGETS_UP**	*Abce1, Acsl1, Ahcy, Aimp2, Ak4, Akap1, Atp1b3, Auh, Bop1, Cad, Cdk4, Cebpz, Ctps1, Ctsc, Cycs, Cyp51, Dancr, Dcun1d4, Ddx10, Ddx21, Dhodh, Ebna1bp2, Exosc2, Exosc7, Fabp5, Fasn, Fkbp4, Fxn, Gcsh, Gpd1l, Grsf1, Hspe1, Iars1, Impdh2, Ldha, Lrp8, Mest, Mgst1, Mrpl3, Mthfd1, Mxi1, Myc, Nampt, Nefh, Nme1, Nolc1, Odc1, Paics, Pebp1, Pno1, Pold2, Polr2h, Ppat, Prdx4, Prps2, Pum3, Pycr1, Rabepk, Ranbp1, Rcc1, Rpia, Rrp1b, Rrs1, Slc16a1, Slc20a1, Slc39a14, Slc39a6, Sord, Srm, Srpk1, Tarbp1, Tbl3, Tfrc, Tmem97, Trap1, Uchl3, Uck2, Vars1, Vrk1, Znf239*
**PENG_RAPAMYCIN_RESPONSE_DN**	*Adrm1, Ahsa1, Aldh4a1, Atf5, Atp5g1, Atp5g3, Bmp1, Btk, Bysl, C8orf41, Calu, Cct5, Cct6a, Cdk4, Chuk, Copb2, Cse1l, Eif2b4, Eif4g2, Emp3, Fam89b, Fasn, Hccs, Hmhb1, Hspa4, Hspa8, Hspe1, Hyou1, Id2, Ifi30, Imp4, Jtb, Lrrc32, Mllt11, Mmp15, Mrpl12, Mrps11, Ndufa9, Npm3, Pfkm, Pgk1, Prmt1, Psmd5, Psmd8, Ptbp1, Rabepk, Rabggtb, Safb, Sf3a3, Sit1, Slc29a1, Snrpc, Sqle, Srm, Tceb3, Tfrc, Tgds, Timm17a, Tmed2, Tmem109, Ube2l3, Wnt10b, Znf259*

Table S2: SeqWell primers.

Primer Name	IDT Order
SeqWell_WTA_Primer (TSO_PCR):	AAGCAGTGGTATCAACGCAGAGT
HTO_WTA_Primer	GTGACTGGAGTTCAGACGTGTGCTCTTCCGATCT

Table S3: Lockdown primers.

Primer Name	IDT Order
IGHM_mouse_lockdown	/5Biosg/CCTTCCCAAATGTCTTCCCCCTCGTCTCCTGCGAGAGCCCCCTGTCTGATAAGAATCTGGTGGCCATGGGCTGCCTIGCCCGGGACTTCC
IGHD_mouse_lockdown	/5Biosg/GAAATCCCACCATCTACCCACTGACACTCCCACGAGCTCTGTCAAGTGACCCAGTGATAATCGGCTGCCTGATTCACGATTACTTCCCTT
IGHA_balb_lockdown	/5Biosg/GTGATAATCGGCTGCCTGATTCACGATTACTTCCCTTICGGCACGATGAATGTGACCTGGGGAAAGAGTGGGAAGGATATAACCACCGT
IGHG12AB_balb_lockdown	/5Biosg/TGCCTGGTCAAGGGITAITTCCCTGAGCCAGTGACIITGACITGGAACTCTGGATCCCTGTCCAGIIGTGTGCACACCTTCCCAGCTITC
IGHG3_balb_lockdown	/5Biosg/CAGCCCCATCTGTCTATCCCTTGGTCCCTGGCTGCGGTGACACATCTGGATCCTCGGTGACACTGGGATGCCTTGTCAAAGGCTACTTCC
IGKC_mouse_lockdown	/5Biosg/TGCACCAACTGTATCCATCTTCCCACCATCCAGTGAGCAGTTAACATCTGGAGGTGCCTCAGTCGTGTGCTTCTTGAACAACTTCTACCC
IGLC_mouse_lockdown	/5Biosg/GTCTTCGCCATCAGTCACCCTGTTTCCACCTTCCTCTGAAGAGCTCGAGACTAACAAGGCCACACTGGTGTGTACGATCACTGATTTCTA
IGLC23_mouse_lockdown	/5Biosg/AGTGGTGTGACAGTGGCCTGGAAGGCAAATGGTACACCTATCACCCAGGGTGTGGACACTTCAAATCCCACCAAAGAGGGCAACAAGTTC

Table S4: V gene primers.

Primer Name	IDT Order	Nextera Seq Handle	5'-3' Seq
mVH01	TCGTGGGCTCGGAGATGTGTATAAGAGACAGCAGGTGCAGCTGCAGCAGCCTGG	TCGTGGGCTCGGAGATGTGTATAAGAGACAG	CAGGTGCAGCTGCAGCAGCCTGG
mVH02	TCGTGGGCTCGGAGATGTGTATAAGAGACAGCAGGTGCAGCTGCAGCAGTCTGG	TCGTGGGCTCGGAGATGTGTATAAGAGACAG	CAGGTGCAGCTGCAGCAGTCTGG
mVH03	TCGTGGGCTCGGAGATGTGTATAAGAGACAGCAGGTGCAGCTGAAGCAGTCTGG	TCGTGGGCTCGGAGATGTGTATAAGAGACAG	CAGGTGCAGCTGAAGCAGTCTGG
mVH04	TCGTGGGCTCGGAGATGTGTATAAGAGACAGCAGGTGCAGCTGAAGGAGTCTGG	TCGTGGGCTCGGAGATGTGTATAAGAGACAG	CAGGTGCAGCTGAAGGAGTCTGG
mVH05	TCGTGGGCTCGGAGATGTGTATAAGAGACAGGAGGTGAAGCTGGAGGAGTCTGG	TCGTGGGCTCGGAGATGTGTATAAGAGACAG	GAGGTGAAGCTGGAGGAGTCTGG
mVH06	TCGTGGGCTCGGAGATGTGTATAAGAGACAGGAGGTGCAGCTGGTGGAGTCTGG	TCGTGGGCTCGGAGATGTGTATAAGAGACAG	GAGGTGCAGCTGGTGGAGTCTGG
mVH07	TCGTGGGCTCGGAGATGTGTATAAGAGACAGGAAGTGCAGCTGTTGGAGACTGG	TCGTGGGCTCGGAGATGTGTATAAGAGACAG	GAAGTGCAGCTGTTGGAGACTGG
mVH08	TCGTGGGCTCGGAGATGTGTATAAGAGACAGGAGGTGCAGCTGCAGCAGTCTGG	TCGTGGGCTCGGAGATGTGTATAAGAGACAG	GAGGTGCAGCTGCAGCAGTCTGG
mVH09	TCGTGGGCTCGGAGATGTGTATAAGAGACAGGAGGTGCAGCTGCAGGAGTCTGG	TCGTGGGCTCGGAGATGTGTATAAGAGACAG	GAGGTGCAGCTGCAGGAGTCTGG
mVH10	TCGTGGGCTCGGAGATGTGTATAAGAGACAGGAGGTGCAGCTGCAGCAGTCTGTG	TCGTGGGCTCGGAGATGTGTATAAGAGACAG	GAGGTGCAGCTGCAGCAGTCTGTG
mVH11	TCGTGGGCTCGGAGATGTGTATAAGAGACAGGAGGTGAAGCTGGTGGAGTCTGG	TCGTGGGCTCGGAGATGTGTATAAGAGACAG	GAGGTGAAGCTGGTGGAGTCTGG
mVH12	TCGTGGGCTCGGAGATGTGTATAAGAGACAGCAGATCCAGCTGCAGCAGTCTGG	TCGTGGGCTCGGAGATGTGTATAAGAGACAG	CAGATCCAGCTGCAGCAGTCTGG
mVH13	TCGTGGGCTCGGAGATGTGTATAAGAGACAGCAGGTTCAGCTGCAACAGTCTGA	TCGTGGGCTCGGAGATGTGTATAAGAGACAG	CAGGTTCAGCTGCAACAGTCTGA
mVH14	TCGTGGGCTCGGAGATGTGTATAAGAGACAGGAGTTCCAGCTGCAGCAGTCTGG	TCGTGGGCTCGGAGATGTGTATAAGAGACAG	GAGTTCCAGCTGCAGCAGTCTGG
mVH15	TCGTGGGCTCGGAGATGTGTATAAGAGACAGGATGTACAGCTTCAGGAGTCAGG	TCGTGGGCTCGGAGATGTGTATAAGAGACAG	GATGTACAGCTTCAGGAGTCAGG

mVH16	TCGTGGGCTCGGAGATGTG TATAAGAGACAGGAGGTGC AGCTTGTTGAGTCTGGTGGA GG	TCGTGGGCTCGGAGA TGTGTATAAGAGACAG	GAGGTGCAGC TTGTTGAGTCT GGTGGAGG
mVH17	TCGTGGGCTCGGAGATGTG TATAAGAGACAGCAGCGTG AGCTGCAGCAGTCTGG	TCGTGGGCTCGGAGA TGTGTATAAGAGACAG	CAGCGTGAGC TGCAGCAGTC TGG
mVH18	TCGTGGGCTCGGAGATGTG TATAAGAGACAGGACGTGA AGCTGGTGGAGTCTGG	TCGTGGGCTCGGAGA TGTGTATAAGAGACAG	GACGTGAAGC TGGTGGAGTC TGG
mVH19	TCGTGGGCTCGGAGATGTG TATAAGAGACAGGAAGTGA TGCTGGTGGAGTCTGG	TCGTGGGCTCGGAGA TGTGTATAAGAGACAG	GAAGTGATGC TGGTGGAGTC TGG
mVH20	TCGTGGGCTCGGAGATGTG TATAAGAGACAGCAGGTGC AGCTTGTAGAGACCGG	TCGTGGGCTCGGAGA TGTGTATAAGAGACAG	CAGGTGCAGC TTGTAGAGAC CGG
mVH21	TCGTGGGCTCGGAGATGTG TATAAGAGACAGCAGATGC AGCTTCAGGAGTCAGG	TCGTGGGCTCGGAGA TGTGTATAAGAGACAG	CAGATGCAGC TTCAGGAGTC AGG
mVH22	TCGTGGGCTCGGAGATGTG TATAAGAGACAGCAGGCTT ATCTACAGCAGTCTGG	TCGTGGGCTCGGAGA TGTGTATAAGAGACAG	CAGGCTTATC TACAGCAGTC TGG
mVH23	TCGTGGGCTCGGAGATGTG TATAAGAGACAGCAGGTCC ARCTGCAGCAGYCTGG	TCGTGGGCTCGGAGA TGTGTATAAGAGACAG	CAGGTCCARC TGCAGCAGYC TGG
mVH24	TCGTGGGCTCGGAGATGTG TATAAGAGACAGGAGGTGA AGCTTCTCSAGTCTGGAGG	TCGTGGGCTCGGAGA TGTGTATAAGAGACAG	GAGGTGAAGC TTCTCSAGTCT GGAGG
mVH25	TCGTGGGCTCGGAGATGTG TATAAGAGACAGCAGGTTA CTCTGAAAGAGTCTGGCC	TCGTGGGCTCGGAGA TGTGTATAAGAGACAG	CAGGTTACTC TGAAAGAGTC TGGCC
mVH26	TCGTGGGCTCGGAGATGTG TATAAGAGACAGCAGGGTC AGATGCAGCAGTCTGG	TCGTGGGCTCGGAGA TGTGTATAAGAGACAG	CAGGGTCAGA TGCAGCAGTC TGG
mVK01	TCGTGGGCTCGGAGATGTG TATAAGAGACAGAACATTA TGATGACACAGTCGCCA	TCGTGGGCTCGGAGA TGTGTATAAGAGACAG	AACATTATGA TGACACAGTC GCCA
mVK02	TCGTGGGCTCGGAGATGTG TATAAGAGACAGAACATTG TGCTGACCCAATCTCCA	TCGTGGGCTCGGAGA TGTGTATAAGAGACAG	AACATTGTGC TGACCCAATC TCCA
mVK03	TCGTGGGCTCGGAGATGTG TATAAGAGACAGCAAATTG TTCTCACCCAGTCTCCA	TCGTGGGCTCGGAGA TGTGTATAAGAGACAG	CAAATTGTTC TCACCCAGTC TCCA
mVK04	TCGTGGGCTCGGAGATGTG TATAAGAGACAGCAAATTG TTCTCTCCCAGTCTCCA	TCGTGGGCTCGGAGA TGTGTATAAGAGACAG	CAAATTGTTC TCTCCCAGTC TCCA

mVK05	TCGTGGGCTCGGAGATGTG TATAAGAGACAGGAAAATG TTCTCACCCAGTCTCCA	TCGTGGGCTCGGAGA TGTGTATAAGAGACAG	GAAAATGTTC TCACCCAGTC TCCA
mVK06	TCGTGGGCTCGGAGATGTG TATAAGAGACAGGAAATTG TGCTCACTCAGTCTCCA	TCGTGGGCTCGGAGA TGTGTATAAGAGACAG	GAAATTGTGC TCACTCAGTC TCCA
mVK07	TCGTGGGCTCGGAGATGTG TATAAGAGACAGGACATCA AGATGACCCAGTCTCCA	TCGTGGGCTCGGAGA TGTGTATAAGAGACAG	GACATCAAGA TGACCCAGTC TCCA
mVK08	TCGTGGGCTCGGAGATGTG TATAAGAGACAGGACATCC AGATGAACCAGTCTCCA	TCGTGGGCTCGGAGA TGTGTATAAGAGACAG	GACATCCAGA TGAACCAGTC TCCA
mVK09	TCGTGGGCTCGGAGATGTG TATAAGAGACAGGACATCC AGATGACTCAGTCTCCA	TCGTGGGCTCGGAGA TGTGTATAAGAGACAG	GACATCCAGA TGACTCAGTC TCCA
mVK10	TCGTGGGCTCGGAGATGTG TATAAGAGACAGGACATTG TGATGACTCAGTCTC	TCGTGGGCTCGGAGA TGTGTATAAGAGACAG	GACATTGTGA TGACTCAGTC TC
mVK11	TCGTGGGCTCGGAGATGTG TATAAGAGACAGGACATTG TGATGTCACAGTCTCCA	TCGTGGGCTCGGAGA TGTGTATAAGAGACAG	GACATTGTGA TGTCACAGTC TCCA
mVK12	TCGTGGGCTCGGAGATGTG TATAAGAGACAGGACATTG TGCTGACCCAATCTCCA	TCGTGGGCTCGGAGA TGTGTATAAGAGACAG	GACATTGTGC TGACCCAATC TCCA
mVK13	TCGTGGGCTCGGAGATGTG TATAAGAGACAGGATATCC AGATGACACAGACTACA	TCGTGGGCTCGGAGA TGTGTATAAGAGACAG	GATATCCAGA TGACACAGAC TACA
mVK14	TCGTGGGCTCGGAGATGTG TATAAGAGACAGGATGTTG TGATGACCCAAACTCCA	TCGTGGGCTCGGAGA TGTGTATAAGAGACAG	GATGTTGTGA TGACCCAAAC TCCA
mVK15	TCGTGGGCTCGGAGATGTG TATAAGAGACAGGAAATCC AGATGACCCAGTCTCCA	TCGTGGGCTCGGAGA TGTGTATAAGAGACAG	GAAATCCAGA TGACCCAGTC TCCA
mVK16	TCGTGGGCTCGGAGATGTG TATAAGAGACAGGACATCC AGATGACACAATCTTCA	TCGTGGGCTCGGAGA TGTGTATAAGAGACAG	GACATCCAGA TGACACAATC TTCA
mVK17	TCGTGGGCTCGGAGATGTG TATAAGAGACAGGACATCC AGATGACCCAGTCTCCA	TCGTGGGCTCGGAGA TGTGTATAAGAGACAG	GACATCCAGA TGACCCAGTC TCCA
mVK18	TCGTGGGCTCGGAGATGTG TATAAGAGACAGGACATCC TGATGACCCAATCTCCA	TCGTGGGCTCGGAGA TGTGTATAAGAGACAG	GACATCCTGA TGACCCAATC TCCA
mVK19	TCGTGGGCTCGGAGATGTG TATAAGAGACAGGACATTG TGCTCACCCAATCTCC	TCGTGGGCTCGGAGA TGTGTATAAGAGACAG	GACATTGTGC TCACCCAATC TCC
mVK20	TCGTGGGCTCGGAGATGTG TATAAGAGACAGGATGTTG	TCGTGGGCTCGGAGA TGTGTATAAGAGACAG	GATGTTGTGG TGACTCAAAC

	TGGTGACTCAAACTCCA		TCCA
mVK21	TCGTGGGCTCGGAGATGTG<br>TATAAGAGACAGAACATTG<br>TAATGACCCAATCTCCC	TCGTGGGCTCGGAGA<br>TGTGTATAAGAGACAG	AACATTGTAA<br>TGACCCAATC<br>TCCC
mVK22	TCGTGGGCTCGGAGATGTG<br>TATAAGAGACAGGATGTTT<br>TGATGACCCAAACTCCA	TCGTGGGCTCGGAGA<br>TGTGTATAAGAGACAG	GATGTTTTGA<br>TGACCCAAAC<br>TCCA
mVK23	TCGTGGGCTCGGAGATGTG<br>TATAAGAGACAGGACATCC<br>AGATGATTCAGTCTCCA	TCGTGGGCTCGGAGA<br>TGTGTATAAGAGACAG	GACATCCAGA<br>TGATTCAGTC<br>TCCA
mVK24	TCGTGGGCTCGGAGATGTG<br>TATAAGAGACAGGACATCT<br>TGCTGACTCAGTCTCCA	TCGTGGGCTCGGAGA<br>TGTGTATAAGAGACAG	GACATCTTGC<br>TGACTCAGTC<br>TCCA
mVK25	TCGTGGGCTCGGAGATGTG<br>TATAAGAGACAGGATGTCC<br>AGATGATTCAGTCTCCA	TCGTGGGCTCGGAGA<br>TGTGTATAAGAGACAG	GATGTCCAGA<br>TGATTCAGTC<br>TCCA
mVK26	TCGTGGGCTCGGAGATGTG<br>TATAAGAGACAGGATGTCC<br>AGATAACCCAGTCTCCA	TCGTGGGCTCGGAGA<br>TGTGTATAAGAGACAG	GATGTCCAGA<br>TAACCCAGTC<br>TCCA
mVK27	TCGTGGGCTCGGAGATGTG<br>TATAAGAGACAGGACATTG<br>TGATGACCCAGTCTCAM	TCGTGGGCTCGGAGA<br>TGTGTATAAGAGACAG	GACATTGTGA<br>TGACCCAGTC<br>TCAM
mVL01	TCGTGGGCTCGGAGATGTG<br>TATAAGAGACAGRGCTGTT<br>GTGACTCAGGAATC	TCGTGGGCTCGGAGA<br>TGTGTATAAGAGACAG	RGCTGTTGTG<br>ACTCAGGAA<br>TC

Table S5: Index PCR primers.

Primer Name	IDT Order	Index Name	P7	Index	Adapter
P7_index_Next	CAAGCAGAAGACGGCATACGAGATGCAGCGTATCGTGGGCTCGGAGATGTG	N721	CAAGCAGAAGACGGCATACGAGAT	GCAGCGTA	TCGTGGGCTCGGAGATGTG
P5_index_TSO01	AATGATACGGCGACCACCGAGATCTACACTAGATCGCGCCTGTCCGCGGAAGCAGTG	N501	AATGATACGGCGACCACCGAGATCTACAC	TAGATCGC	GCCTGTCCGCGGAAGCAGTG
P5_index_TSO02	AATGATACGGCGACCACCGAGATCTACACCTCTCTATGCCTGTCCGCGGAAGCAGTG	N502	AATGATACGGCGACCACCGAGATCTACAC	CTCTCTAT	GCCTGTCCGCGGAAGCAGTG
P5_index_TSO03	AATGATACGGCGACCACCGAGATCTACACTATCCTCTGCCTGTCCGCGGAAGCAGTG	N503	AATGATACGGCGACCACCGAGATCTACAC	TATCCTCT	GCCTGTCCGCGGAAGCAGTG
P5_index_TSO04	AATGATACGGCGACCACCGAGATCTACACAGAGTAGAGCCTGTCCGCGGAAGCAGTG	N504	AATGATACGGCGACCACCGAGATCTACAC	AGAGTAGA	GCCTGTCCGCGGAAGCAGTG
P5_index_TSO05	AATGATACGGCGACCACCGAGATCTACACGTAAGGAGGCCTGTCCGCGGAAGCAGTG	N505	AATGATACGGCGACCACCGAGATCTACAC	GTAAGGAG	GCCTGTCCGCGGAAGCAGTG
P5_index_TSO06	AATGATACGGCGACCACCGAGATCTACACACTGCATAGCCTGTCCGCGGAAGCAGTG	N506	AATGATACGGCGACCACCGAGATCTACAC	ACTGCATA	GCCTGTCCGCGGAAGCAGTG
P5_index_TSO07	AATGATACGGCGACCACCGAGATCTACACCTAAGCCTGCCTGTCCGCGGAAGCAGTG	N508	AATGATACGGCGACCACCGAGATCTACAC	CTAAGCCT	GCCTGTCCGCGGAAGCAGTG
P5_index_TSO08	AATGATACGGCGACCACCGAGATCTACACCGTCTAATGCCTGTCCGCGGAAGCAGTG	N510	AATGATACGGCGACCACCGAGATCTACAC	CGTCTAAT	GCCTGTCCGCGGAAGCAGTG
P5_index_TSO09	AATGATACGGCGACCACCGAGATCTACACTCTCTCCGGCCTGTCCGCGGAAGCAGTG	N511	AATGATACGGCGACCACCGAGATCTACAC	TCTCTCCG	GCCTGTCCGCGGAAGCAGTG
P5_index_TSO10	AATGATACGGCGACCACCGAGATCTACACTCGACTAGGCCTGTCC	N513	AATGATACGGCGACCACCGAGATCTACAC	TCGACTAG	GCCTGTCCGCGGAAGCAGTG

	GCGGAAGCAGTG				
P5_index_TSO11	AATGATACGGCGACC<br>ACCGAGATCTACACT<br>TCTAGCTGCCTGTCC<br>GCGGAAGCAGTG	N515	AATGATACGGC<br>GACCACCGAGA<br>TCTACAC	TTCTAGCT	GCCTGTC<br>CGCGGAA<br>GCAGTG
P5_index_TSO12	AATGATACGGCGACC<br>ACCGAGATCTACACC<br>CTAGAGTGCCTGTCC<br>GCGGAAGCAGTG	N516	AATGATACGGC<br>GACCACCGAGA<br>TCTACAC	CCTAGAGT	GCCTGTC<br>CGCGGAA<br>GCAGTG
P5_index_TSO13	AATGATACGGCGACC<br>ACCGAGATCTACACG<br>CGTAAGAGCCTGTCC<br>GCGGAAGCAGTG	N517	AATGATACGGC<br>GACCACCGAGA<br>TCTACAC	GCGTAAGA	GCCTGTC<br>CGCGGAA<br>GCAGTG
P5_index_TSO14	AATGATACGGCGACC<br>ACCGAGATCTACACA<br>AGGCTATGCCTGTCC<br>GCGGAAGCAGTG	N520	AATGATACGGC<br>GACCACCGAGA<br>TCTACAC	AAGGCTAT	GCCTGTC<br>CGCGGAA<br>GCAGTG
P5_index_TSO15	AATGATACGGCGACC<br>ACCGAGATCTACACG<br>AGCCTTAGCCTGTCC<br>GCGGAAGCAGTG	N521	AATGATACGGC<br>GACCACCGAGA<br>TCTACAC	GAGCCTTA	GCCTGTC<br>CGCGGAA<br>GCAGTG
P5_index_TSO16	AATGATACGGCGACC<br>ACCGAGATCTACACT<br>TATGCGAGCCTGTCC<br>GCGGAAGCAGTG	N522	AATGATACGGC<br>GACCACCGAGA<br>TCTACAC	TTATGCGA	GCCTGTC<br>CGCGGAA<br>GCAGTG

Table S6: Sequencing primers.

Primer Name	IDT Order
IGHM_mouse_seq	GCTCTCGCAGGAGACGAGGGGGAAGACATTTGGGAA
IGHD_mouse_seq	GGGCTTTGCACTCTGAGAGGAGGAACATGTCAG
IGHG12AB_mouse_seq	CAGGGGCCAGTGGATAGACIGATGGGGITGT
IGHG3_mouse_seq	CCGAGGATCCAGATGTGTCACCGCAGCCAGGG
IGHA_mouse_seq	AGGACTGGTGGGAGTGTCAGTGGGTAGATGGTGGGAT
IGLC1_mouse_seq	TCTTCAGAGGAAGGTGGAAACAGGGTGACTGATGGCGAA
IGLC2_mouse_seq	CTCAGAGGAAGGTGGAAACAIGGTGAGIGTGGGAGTGG
IGKC_mouse_seq	TCCAGATGTTAACTGCTCACTGGATGGTGGGAAGATGGA TACAGTTGGTG
SeqWell Read 1	GCCTGTCCGCGGAAGCAGTGGTATCAACGCAGAGTAC
SeqWell_Index_Read2_primer	ACTCTGCGTTGATACCACTGCTTCCGCGGACAGGC

Data file S1. Individual-level data.
